# Discovery and *In Silico* Characterization of Anatolian Water Buffalo Rumen-Derived Bacterial Thermostable Xylanases: A Sequence-Based Metagenomic Approach

**DOI:** 10.1021/acsomega.5c00965

**Published:** 2025-03-18

**Authors:** Halil Kurt, Dilek Sever Kaya, İsmail Akçok, Ceyhun Sarı, Ebru Albayrak, Hasan Murat Velioğlu, Hasan Ersin Şamlı, Mehmet Levent Özdüven, Yusuf Sürmeli

**Affiliations:** 1 Department of Medical Biology, Hamidiye International School of Medicine, University of Health Sciences, Istanbul 34668, Turkey; 2 Clinical Nutrition and Microbiota Research Laboratory, Istanbul Faculty of Medicine, Istanbul University, İstanbul 34390, Turkey; 3 Department of Bioengineering, Faculty of Life and Natural Sciences, Abdullah Gul University, Kayseri 38080, Turkey; 4 Department of Agricultural Biotechnology, Faculty of Agriculture, Tekirdag Namik Kemal University, Tekirdag 59030, Turkey; 5 Department of Animal Science, Faculty of Agriculture, Tekirdag Namik Kemal University, Tekirdag 59030, Turkey

## Abstract

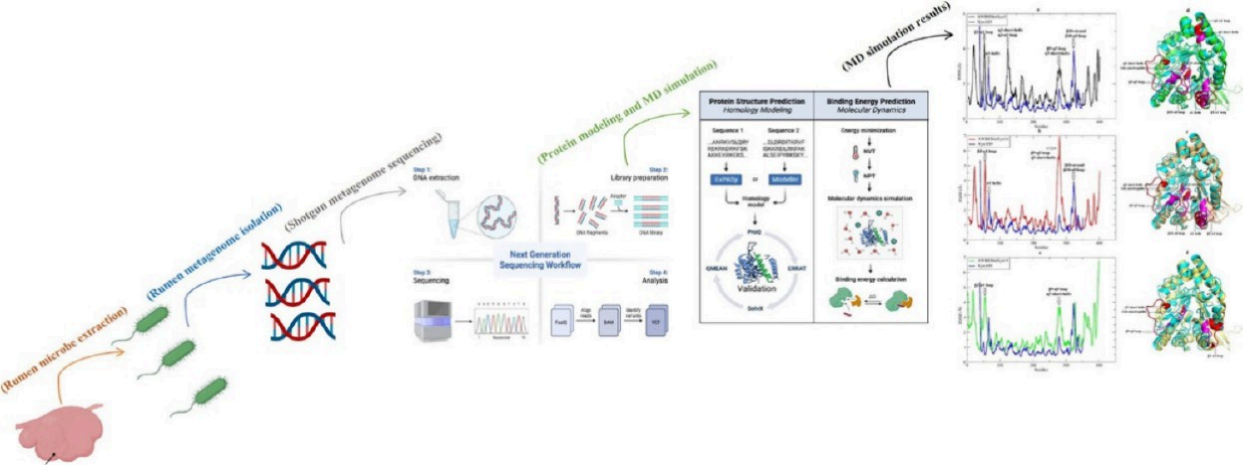

This study involved shotgun sequencing of rumen metagenomes from three Anatolian water buffalos, an exploration of the relationship between microbial flora and xylanases, and *in silico* analyses of thermostable xylanases, focusing on their sequence, structure, and dynamic properties. For this purpose, the rumen metagenome of three Anatolian water buffalos was sequenced and bioinformatically analyzed to determine microbial diversity and full-length xylanases. Analyses of BLAST, biophysicochemical characteristics, phylogenetic tree, and multiple sequence alignment were performed with Blastp, ProtParam, MEGA11 software, and Clustal Omega, respectively. Three-dimensional homology models of three xylanases (AWBRMetXyn5, AWBRMetXyn10, and AWBRMetXyn19) were constructed by SWISS-MODEL and validated by ProSA, ProCheck, and Verify3D. Also, their 3D models were structurally analyzed by PyMOL, BANΔIT, thermostability predictor, What If, and Protein Interaction Calculator (PIC) software. Protein–ligand interactions were examined by docking and MD simulation. Shotgun sequence and Blastp analyses showed that *Clostridium* (Clostridiales bacterial order), *Ruminococcus* (Oscillospiraceae bacterial family), *Prevotella* (Bacteroidales bacterial order), and *Butyrivibrio* (Lachnospiraceae bacterial family) were found as dominant potential xylanase-producer genera in three rumen samples. Furthermore, the biophysicochemical analysis indicated that three xylanases exhibited an aliphatic index above 80, an instability index below 40, and melting temperatures (*T*_m_) surpassing 65 °C. Phylogenetic analysis placed three xylanases within the GH10 family, clustering them with thermophilic xylanases, while homology modeling identified the optimal template as a xylanase from a thermophilic bacterium. The structural analysis indicated that three xylanases possessed the number of salt bridges, hydrophobic interactions, and *T*_m_ score higher than 50, 165, and 70 °C, respectively; however, the reference thermophilic XynAS9 had 43, 145, and 54.41 °C, respectively. BANΔIT analysis revealed that three xylanases exhibited lower *B*′-factor values in the β3-α1 loop/short-helix at the N-terminal site compared to the reference thermophilic XynAS9. In contrast, six residues (G79, M123, D150, T199, A329, and G377) possessed higher *B*′-factor values in AWBRMetXyn5 and their aligned positions in AWBRMetXyn10 and AWBRMetXyn19, relative to XynAS9 including Gln, Glu, Ile, Lys, Ser, and Val at these positions, respectively. MD simulation results showed that the β9-η5 loop including catalytic nucleophile glutamic acid in the RMSF plot of three xylanases had a higher fluctuation than the aligned region in XynAS9. The distance analysis from the MD simulation showed that the nucleophile residue in AWBRMetXyn5 and AWBRMetXyn10 remained closer to the ligand throughout the simulation compared with XynAS9 and AWBRMetXyn19. The most notable difference between AWBRMetXyn5 and AWBRMetXyn10 was the increased amino acid fluctuations in two specific regions, the η3 short-helix and the η3-α3 loop, despite a minimal sequence difference of only 1.24%, which included three key amino acid variations (N345, N396, and T397 in AWBRMetXyn5; D345, K396, and A397 in AWBRMetXyn10). Thus, this study provided computational insights into xylanase function and thermostability, which could inform future protein engineering efforts. Additionally, three xylanases, especially AWBRMetXyn5, are promising candidates for various high-temperature industrial applications. In a forthcoming study, three xylanases will be experimentally characterized and considered for potential industrial applications. In addition, the amino acid substitutions (G79Q, M123E, D150I, T199K, A329S, and G377V) and the residues in the β3-α1 loop will be targeted for thermostability improvement of AWBRMetXyn5. The amino acids (N345, N396, and T397) and the residues on the β9-η5 loop, η3 short-helix, and η3-α3 loop will also be focused on development of the catalytic efficiency.

## 1 Introduction

Lignocellulose, a valuable and untapped renewable carbon source, primarily consists of cellulose, hemicellulose, and lignin.^1−3^ Hemicellulose, mainly xylan, consists of a β-1,4-linked xylopyranose framework with β-1,3-linked l-arabinose and α-1,2-linked d-glucopyranose branches.^4,5^ Xylanases (3.2.1.8) break down xylan by randomly hydrolyzing β-1,4-glycosidic bonds in its backbone, targeting xylopyranosyl residues.^6^ They are categorized into different groups based on the amino acid sequences found in their catalytic domains. These groups are represented by nine glycoside hydrolase (GH) families (GH5, GH8, GH10, GH11, GH30, GH43, GH51, GH98, and GH141), which are documented in the Carbohydrate Active Enzymes (CAZy) database^7^ (http://www.cazy.org/).

The xylanase market has grown steadily for three decades, attracting interest in fruit juice enrichment and animal feed applications, which require high temperatures.^8−13^ These enzymes must exhibit stability under harsh industrial conditions, including high temperatures.^14^ As a result, there is a growing demand for thermostable xylanases in various industrial processes driven by their extensive biotechnological applications.

Xylanases are obtained from various sources, especially the rumen of ruminants, where plant polysaccharides are efficiently broken down.^15^ The rumen, which constitutes 55% of the total stomach of ruminants, serves as the primary site for microbial fermentation of plant lignocellulosic material.^16^ The rumen ecosystem is a complex structure that harbors various microorganisms including obligate anaerobes, bacteria, fungi, protozoa, and archaea. These microorganisms make the rumen a promising source of hydrolytic enzymes, including xylanases.^15,17,18^ Nevertheless, more than 85% of rumen microorganisms cannot be cultured, leaving a substantial number of functional genes yet to be identified.^19^ The metagenomic approach enables cultivation-independent screening to discover new biocatalysts, including xylanases, from environments like the rumen.^9^ This approach has been utilized in studies involving Hu sheep,^20^ goat,^21^ cow,^22^ ox,^23^ cattle,^24−26^ and camel^14,17^ to identify and characterize thermostable xylanases.

Buffalos (*Bubalus bubalis*) can thrive on low-quality roughage, agricultural residues, and industrial waste rich in lignocellulose, enabling proper growth and nutrition.^27^ Consequently, the rumen of buffalos serves as an environmental niche abundant in lignocellulolytic enzymes including xylanases. Notably, Anatolian water buffalo exhibited superior lignocellulose digestion compared to cattle calves, and their rumen harbors a greater diversity of bacterial species.^28^ In line with this, a study demonstrated that buffalo rumen possesses a higher population of cellulolytic bacteria compared to bovine rumen.^29^ Despite these findings, research on xylanase enzymes derived from buffalo rumen is limited.^30−32^ To our knowledge, no investigation on xylanase enzymes derived from the Anatolian water buffalo rumen has been conducted thus far. This study aimed to perform the exploration and computational characterization of Anatolian water buffalo rumen-derived bacterial xylanases by a sequence-based metagenomic approach. The findings were compared and analyzed with previously studied xylanases described in the literature.

## 2 Materials and Methods

### 2.1 Sampling from Rumen Digesta and Metagenomic DNA Extraction

Rumen digesta were collected from three mature male Anatolian water buffalos, freshly slaughtered and aged 2.5, 2, and 4.5, named YR1, YR2, and YR3, respectively. The solid and liquid fractions of the rumen digests were transferred to sterile containers. The fractions were kept on ice during transfer to the laboratory and stored at −86 °C until the metagenomic DNA isolation. Metagenomic bacterial DNA isolation from three rumen digesta was performed using a fecal DNA isolation kit (QIAamp PowerFecal DNA isolation kit), according to the manufacturer’s instructions.

### 2.2 Metagenome Sequencing, Bioinformatics Analysis, and Gene Finding

Sequencing of metagenomic DNA samples of YR1, YR2, and YR3 was performed with an Illumina NextSeq 550 next-generation sequencing platform. For this purpose, the genomic DNA library was prepared by adjusting the Nextera DNA Flex kit (Illumina) to 100 ng quantities based on the manufacturer’s instructions. The DNA samples were sequenced by using the Illumina NextSeq 550 next-generation sequencing platform. To ensure the quality of the obtained reads in fastq format, the FASTQC program^33^ was used for quality control, and any poor readings and barcodes were eliminated using the Trimmomatic program.^34^ Approximately 300 base pairs of long reads were constructed by merging the high-quality paired reads obtained in the previous step with the assistance of MOTHUR.^35^ These reads were then compared to the NCBI nr protein database using the Diamond program^36^ through BLASTx analysis. The taxonomic and functional analyses of the BLAST results were conducted using the Megan6 program.^37^ Metagenome assembly was performed using the metasensitive presets in the Megahit program.^38^ The genes were predicted by the Prokka program in metagenome assemble reads.^39^ Then, xylanase ORFs were downloaded from the NCBI protein database, and a database was created from the protein sequences by using the Diamond program. BLAST searches were performed using diamond for metagenome sequences and contigs obtained from genome assembly. Xylanase genes were identified based on reads with an *E*-value less than 10^–5^ and at least 80% sequence similarity. The functional analysis of the selected sequences was performed using the PROSITE program.^40^ The amino acid sequences of the xylanases from the rumen of Anatolian water buffalo were used to determine the closest amino acid sequence and its microbial source by the BLASTp tool.^41,42^

### 2.3 Biophysicochemical Characteristics and Phylogenetic Relationships of the Xylanases

The biophysicochemical characteristics of the bacterial xylanases were investigated using xylanase amino acid sequences by the ProtParam tool^43^ and *T*_m_ predictor.^44^ For this purpose, the theoretical pI value, instability index, grand average of the hydropathicity index (GRAVY), aliphatic index, aa length, molecular mass (kDa), and theoretical *T*_m_ ranges were determined. Also, the molecular phylogeny analysis of the xylanases with the other characterized xylanases was performed by MEGA11 software. To accomplish this, the analysis was conducted using the UPGMA statistical method with 1000 bootstrap replications using various GH family xylanases.^45^ The 25 representative enzymes from the existing six GH families were selected from the CAZy database to determine the phylogenetic relationship of the enzymes.^7^ Enzymes with a *T*_m_ value above 65 °C and an aliphatic index score above 80 were considered potential thermostable xylanases, and further analyses were conducted with these enzymes.

### 2.4 Homology Modeling

The predicted model structures of the xylanases were built by ProMod3 software in the SWISS-MODEL online homology model server.^46−49^ The cross-validation of the xylanases was carried out by different bioinformatics programs. Regarding this, ProSA was utilized to evaluate the local and the global quality levels by determination of the *Z* scores.^50^ To analyze the stereochemical qualities and dihedral angles of the xylanase models, the Ramachandran plot was formed via ProCheck.^51^ Based on the known structure, the prediction of compatibility between xylanase models and their amino acid sequences was performed by Verify3D.^52^

### 2.5 Thermostability and Noncovalent Interaction Analyses

The thermostability of the xylanase models was predicted by the BANΔIT^53^ and thermostability predictor^54^ programs. For BANΔIT analysis, the pdb files of the xylanase models were submitted to the program, and *B*′-factor distribution was determined for each amino acid of the enzyme models. The lower the *B*′-factor, the higher the rigidity indicating higher stability of the enzymes. In addition, thermostability analysis of the xylanase models was estimated by a thermostability predictor by giving predicted *T*_m_ scores. Also, salt bridges of the enzymes were calculated up to 4 Å by the What If server.^55^ The hydrophobic interactions were determined by the Protein Interaction Calculator (PIC) server.^56^

### 2.6 Molecular Docking Analysis

Molecular docking of xylanase models was carried out to study their interactions with various xylooligosaccharides, including 26 compounds sourced from PubChem such as xylobiose (X_2_), xylotriose (X_3_), xylotetraose (X_4_), xylopentaose (X_5_), xylohexaose (X_6_), xyloheptaose (X_7_), and others. The docking was performed using Smina, a modified version of AutoDock Vina (v1.1.2).^57,58^ Ligand files were converted to ready-to-dock format by using OpenBabel version 3.1.1.^57^ The center coordinates of 51.84, 26.22, and 123.71 with dimensions of 33.04, 38.16, and 18.06 were used for the GH10 xylanase models. For XynAS9, center coordinates of 20.17, −17.20, and −13.69, along with sizes of 23.83, 32.79, and 23.41, respectively, were applied. Interactions were visualized using LigPlot+ and PyMOL.^59^

### 2.7 Molecular Dynamics (MD) Simulation

Molecular dynamics (MD) simulations were used to investigate the interactions between the enzymes and the xylotetraose (X_4_) ligand and to show the stability of the docked complexes throughout 300 ns. For this purpose, each protein was prepared for molecular dynamics (MD) simulation using Chimera’s DockPrep tool, which adds missing hydrogen atoms and assigns charges.^60,61^ Ligand topologies for the OPLS-AA force field were generated using the LigParGen web server, which was developed by the same group that introduced the OPLS-AA force field.^62−65^ The MD simulation was set to maintain a temperature of 300 K (37 °C) during the first 100 ns following NVT and NPT equilibration. The temperature was then linearly increased from 300 K (37 °C) to 339 K (66 °C) between 100 and 200 ns and then fixed at 339 K (66 °C) until 300 ns. This simulation protocol was executed using GROMACS version 2021.^66,67^ All simulations were performed by using the OPLS-AA force field. The protein–ligand complexes were placed in a triclinic box containing the TIP3P water model, with 17,539 water molecules and appropriate NaCl molecules to neutralize the system at a concentration of 0.1 M.^64^ After generating the box, energy minimization was carried out using the steepest descent method with a maximum of 5000 steps. This was followed by a 100 ps NVT simulation using a V-rescale thermostat and then a 200 ps NPT simulation with a linear temperature increase from 0 to 300 K, utilizing Berendsen pressure coupling.^68,69^ The equilibrated systems underwent a 300 ns simulation using the previously mentioned temperature settings, with a 2 fs time step and energy and coordinate data saved every 10 ps. During the simulation, a pressure of 1 bar was maintained using a Parrinello–Rahman barostat.^70^ Covalent bond constraints for these systems were applied using LINCS, while particle mesh Ewald (PME) was employed to calculate long-range electrostatic interactions with a cutoff of 1.2 nm.^71,72^ Finally, root-mean-square deviation (RMSD), root-mean-square fluctuation (RMSF), and distance analyses were conducted to shed light on the dynamic behavior of the enzymes.

### 2.8 Data Representation

The figures were visualized by GraphPad Prism version 6.00 for Windows (GraphPad Software, La Jolla, CA, USA) (www.graphpad.com), PyMOL Molecular Graphics System, version 2.0 (Schrödinger, LLC), and MEGA11 software.

### 2.9 Potential Future Experimental Setup

In potential future studies, the enzymes will be cloned via recombinant DNA technology and heterologously expressed. They will undergo purification through chromatographic techniques, followed by characterization, including determination of optimal temperature, optimal pH, thermal stability, pH stability, catalytic activity, and kinetic parameters. Moreover, the amino acid substitutions identified in this study will be experimentally investigated for their impact on thermostability and catalytic efficiency, utilizing state-of-the-art strategies from rational and semirational protein engineering approaches.

## 3 Results

The metagenomic discovery and computational characterization of thermostable xylanases were carried out using the rumen digesta of three Anatolian water buffalos (YR1, YR2, and YR3). For this purpose, rumen metagenomes of three samples were sequenced and bioinformatically analyzed to determine their microbial diversity and full-length xylanase candidates. The closest amino acid sequences, biophysicochemical characteristics, and phylogenetic relationships were determined for 19 full-length xylanase amino acid sequences. Then, three GH10 bacterial xylanases (AWBRMetXyn5, AWBRMetXyn10, and AWBRMetXyn19), which might be determined as highly thermostable enzymes, were computationally characterized in terms of the 3D predicted structures, thermostability capacity of the 3D models, conserved amino acids, and protein–ligand interactions by molecular docking and MD simulation.

### 3.1 Bioinformatics Analysis of Anatolian Water Buffalo Rumen Metadata

Illumina next-generation sequencing was employed to investigate the microbial diversity in rumen samples from Anatolian buffalos. A mean of 20,027,793 ± 2,054,449 reads was generated, with 98.9% remaining after quality filtering. From these, an average of 19,808,455 ± 2,041,273 high-quality reads (>Q30) underwent de novo assembly using the Megahit De Novo Assembly tool, resulting in contigs with a total length of 731,069,956 ± 67,513,184 bp and an *L*_50_ length of 448,622 ± 68,929 bp. An average of 39,627 ± 12,771 genes within the assembled contigs were identified, and gene prediction can be found in Table S1.

**Figure 1:**
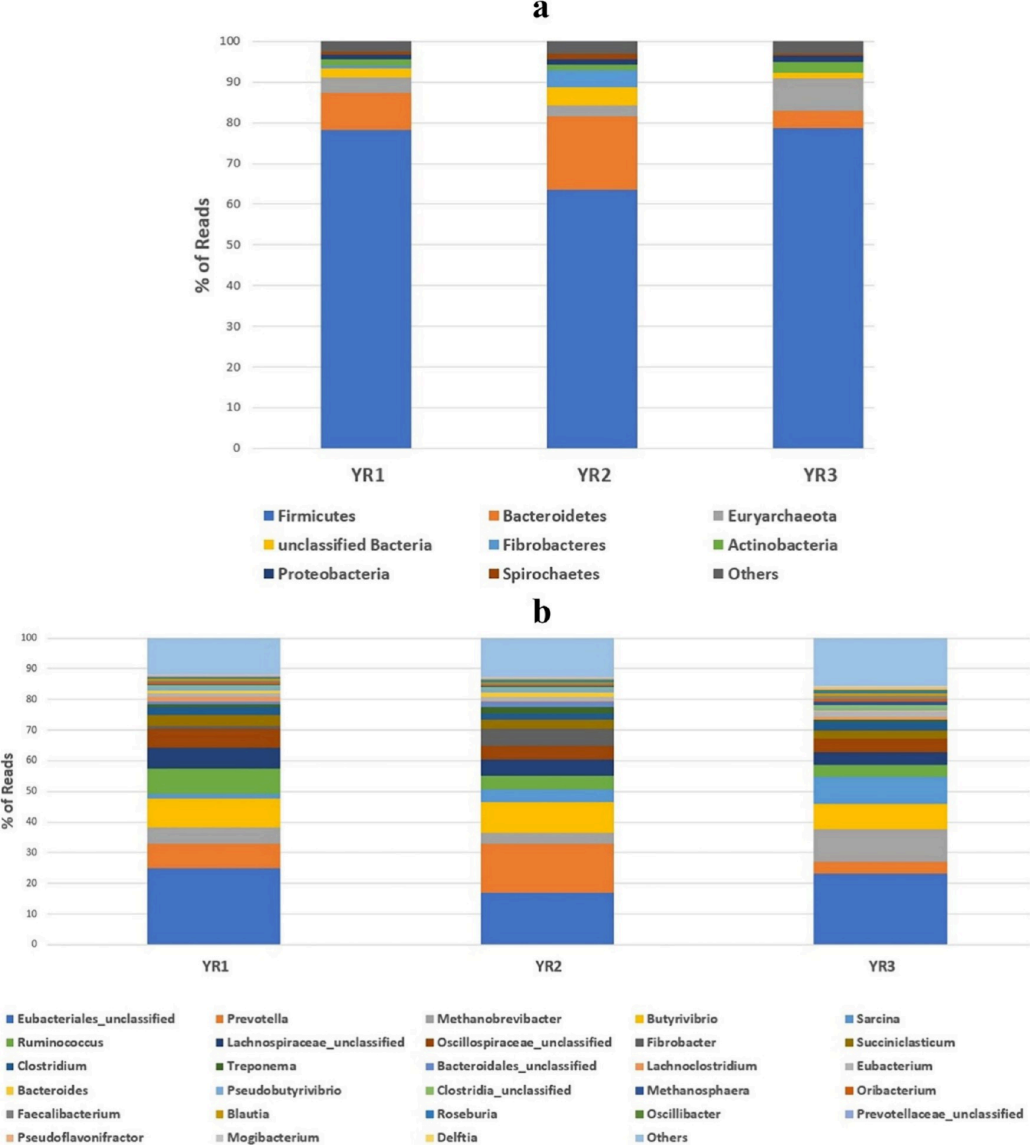
Microbial diversity of rumens of three Anatolian water buffalos based on metagenomic shotgun sequencing. (a) Phylum level diversity and (b) genus level diversity.

Anatolian water buffalo rumen metagenome-derived sequences were identified, and their taxonomic diversity was analyzed at the phylum and genera level. The analysis showed that the majority of organisms, comprising over 92%, were identified as bacteria, making them the most prevalent domain across all the samples. Among bacteria, the most abundant phylum was determined as Firmicutes, followed by Bacteroidetes. The combined total of these two accounted for over 80% of the overall microorganism population in all samples (Figure 1a). The present work also indicated that a higher abundance of genera was *Prevotella*, *Methanobrevibacter*, *Butyrivibrio*, *Sarcina*, *Ruminococcus*, *Fibrobacter*, *Succiniclasticum*, and *Clostridium*, which were found by about 40, 50, and 40% in YR1, YR2, and YR3, respectively. A notable proportion of the remaining segment was attributed to the unclassified Eubacteriales order and unclassified families (Lachnospiraceae and Oscillospiraceae) (Figure 1b).

In this study, rumen metagenome data from three Anatolian buffalos revealed the presence of lignocellulose-degrading enzymes. Accordingly, the rumen metagenome included 2941 xylanolytic enzyme sequence reads, predominantly endo-1,4-β-xylanases, α-d-glucuronidases, β-mannanases, α-l-arabinofuranosidases, and β-xylosidases. Additionally, 1338 cellulolytic enzyme sequences, including endocellulases/endoglucanases and exocellulases/cellobiohydrolases, as well as 438 pectinolytic enzymes, mostly endo/exopolygalacturonases and pectin methyl esterases, were determined (Table S2).

Among xylanase sequences, 19 full-length xylanases from the Anatolian water buffalo rumen were determined and then analyzed to assign the closest amino acid sequence by BLASTp. The analysis findings indicated that the majority of the closely related sequences were associated with the Clostridiales bacterial order, and the other xylanases were related to the Oscillospiraceae bacterial family, Bacteroidales bacterial order, Anaerolineaceae bacterial family, and Lachnospiraceae bacterial family (Table S3).

### 3.2 The Analysis of Biophysicochemical Aspects of the Xylanases

Various biophysicochemical properties (theoretical pI, molecular weight, aa length, instability index, GRAVY, aliphatic index, and *T*_m_) of 19 full-length bacterial xylanase sequences from the Anatolian water buffalo rumen were investigated at the sequence level by the ProtParam tool and *T*_m_ predictor program. The results indicated that most of the sequences (except AWBRMetXyn9 and AWBRMetXyn13) had a pI value in the range of 4.30–5.31. In addition, *T*_m_ predictor analysis showed that most of the xylanase sequences possessed higher *T*_m_ than 55 °C. Among these, three xylanases (AWBRMetXyn5, AWBRMetXyn10, and AWBRMetXyn19) had a bigger *T*_m_ value than 65 °C. The biophysicochemical analysis results also showed that the aliphatic index and the instability index values were found in the ranges of 53.99–87.22 and 20.30–48.17, respectively. The aliphatic index values of AWBRMetXyn5, AWBRMetXyn10, and AWBRMetXyn19 were above 80, whereas their instability index was found to be 31–39 (Table 1). In addition, ranges of the instability and aliphatic index of biochemically characterized bacterial thermostable xylanases from *Streptomyces* sp*.* S9, *Bacillus halodurans* S7, and *Anoxybacillus sp.* E2 were found to be 30–39 and 73–85, respectively. In addition, the instability and aliphatic indexes of fungal GH11 thermostable xylanase from *Thermomyces lanuginosus* were found to be 26.14 and 62.04, respectively (Table S4).

**Table 1:** Biophysicochemical Aspects of the 19 Full-Length Bacterial Xylanases from the Rumen of the Anatolian Water Buffalo

**no.**	**protein ID**	**protein name**	**theoretical pI**	**instability index**	**GRAVY**^a^	**aliphatic index**	**aa length**	**molecular weight (kDa)**	*T*_**m**_**(°C)**	**family**^b^
1	DIANCIHN_00801	AWBRMetXyn1	5.27	36.08	–0.628	73.38	388	46.42	55–65	GH10
2	DIANCIHN_01743	AWBRMetXyn2	4.30	34.00	–0.281	81.26	715	78.86	55–65	GH10
3	DIANCIHN_02209	AWBRMetXyn3	5.60	33.87	–0.643	53.99	311	36.40	<55	GH8
4	DIANCIHN_11883	AWBRMetXyn4	4.82	43.51	–0.443	64.47	376	43.24	<55	GH8
5	DIANCIHN_13666	AWBRMetXyn5	5.05	31.46	–0.374	82.73	403	45.82	>65	GH10
6	DIANCIHN_33359	AWBRMetXyn6	4.97	48.17	–0.436	64.20	376	43.34	<55	GH8
7	DIANCIHN_39032	AWBRMetXyn7	5.31	32.98	–0.373	81.63	410	46.54	55–65	GH10
8	DIANCIHN_41040	AWBRMetXyn8	4.43	32.60	–0.199	78.61	675	73.92	55–65	GH10
9	DIANCIHN_44290	AWBRMetXyn9	7.70	22.46	–0.280	77.96	368	40.94	<55	GH30
10	MLOJOCKJ_01271	AWBRMetXyn10	5.06	32.43	–0.371	82.98	403	45.80	>65	GH10
11	MLOJOCKJ_01367	AWBRMetXyn11	5.21	34.22	–0.368	84.24	410	46.54	55–65	GH10
12	MLOJOCKJ_03719	AWBRMetXyn12	4.64	29.28	–0.298	74.99	674	74.41	55–65	GH10
13	MLOJOCKJ_07132	AWBRMetXyn13	8.21	35.02	–0.254	78.25	359	39.84	55–65	GH30
14	MLOJOCKJ_14190	AWBRMetXyn14	5.14	30.56	–0.659	71.78	387	46.17	55–65	GH10
15	MLOJOCKJ_18013	AWBRMetXyn15	5.27	36.08	–0.628	73.38	388	46.42	55–65	GH10
16	MLOJOCKJ_21568	AWBRMetXyn16	4.31	20.30	–0.468	56.26	540	59.55	55–65	GH5
17	ELKBKCFI_13369	AWBRMetXyn17	5.27	37.14	–0.620	73.38	388	46.46	55–65	GH10
18	ELKBKCFI_26736	AWBRMetXyn18	4.76	26.99	–0.542	66.29	596	67.53	55–65	GH43
19	ELKBKCFI_43865	AWBRMetXyn19	5.13	39.76	–0.329	87.22	406	46.13	>65	GH10

a Grand average of the hydropathicity index.

b GH families of xylanases obtained from the phylogenetic tree.

### 3.3 Evolutionary Relationship of the Xylanases from the Rumen of the Anatolian Water Buffalo

**Figure 2:**
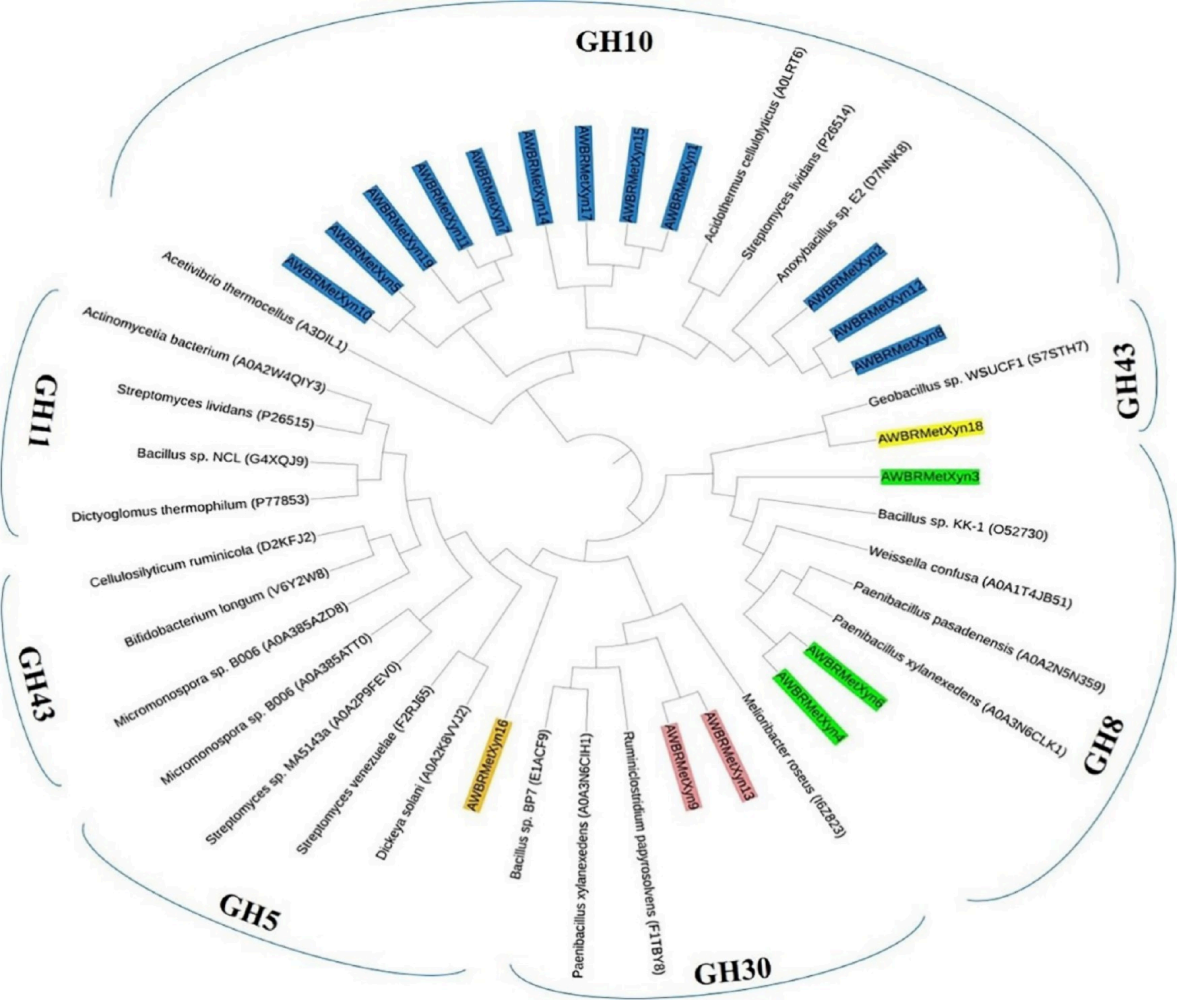
Phylogenetic tree of the 19 full-length xylanases from the rumen of the Anatolian water buffalo and different members of various GH family xylanases. The UniProt accession numbers and the names of the microorganisms were provided. GH5, GH8, GH10, GH30, and GH43 family members were highlighted by orange, green, blue, salmon, and yellow colors, respectively.

The evolutionary relationship of 19 full-length bacterial xylanases from the rumen of the Anatolian water buffalo was investigated by MEGA 11 with the UPGMA method. The analysis showed that the xylanases were distributed into five families (GH5, GH8, GH10, GH30, and GH43). Twelve xylanases (AWBRMetXyn1, 2, 5, 7, 8, 10, 11, 12, 14, 15, 17, and 19) were clustered within the GH10 family. In addition, AWBRMetXyn18 was grouped into a GH43 family and AWBRMetXyn16 was placed in a group of GH5 family (Figure 2). Also, two xylanases AWBRMetXyn9 and AWBRMetXyn13 were placed in a cluster of the GH30 family, and the others (AWBRMetXyn3, AWBRMetXyn4, and AWBRMetXyn6) were the closest to those in the GH8 family. Among all, AWBRMetXyn5, AWBRMetXyn10, and AWBRMetXyn19 were chosen for further analyses since they possessed a *T*_m_ value exceeding 65 °C.

### 3.4 Amino Acid Sequence Alignment of the Three Xylanases

**Figure 3:**
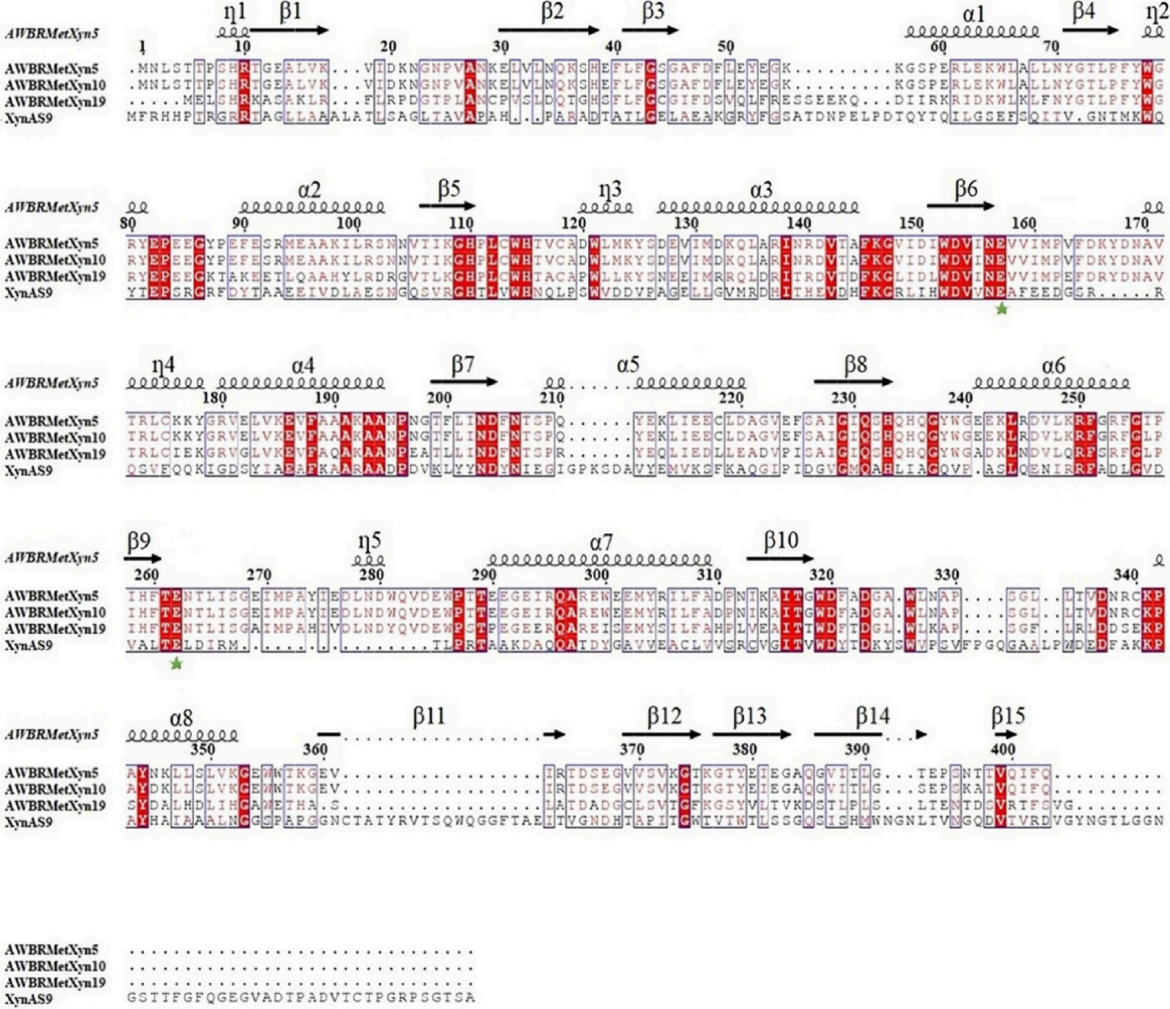
Amino acid sequence alignment of the three bacterial xylanases from Anatolian water buffalo relative to thermophilic reference xylanase (XynAS9). The red background is used to highlight strictly conserved residues, while conservatively substituted residues are boxed. The secondary structural elements of AWBRMetXyn5, α-helix (α), β-strand (β), and short-helix (η), are displayed above the aligned sequences. The conserved catalytic residues (E157 and E262) are marked with green asterisks. The figure was generated using ESPript.^73^

The sequence alignment of AWBRMetXyn5, AWBRMetXyn10, and AWBRMetXyn19 was analyzed by using Clustal Omega. These enzymes were compared to the thermophilic reference XynAS9. According to the alignment findings, the amino acid sequence of AWBRMetXyn19 exhibited 60.30 and 60.80% similarity to those of AWBRMetXyn5 and AWBRMetXyn10, respectively. On the other hand, the sequence of AWBRMetXyn5 possessed high similarity with that of AWBRMetXyn10 by 98.76%, and they had differences in only five amino acids (I256, N345, T392, N396, and T397 for AWBRMetXyn5 and L256, D345, S392, K396, and A397 for AWBRMetXyn10). In addition, many conserved residues including two catalytic amino acids (E157 and E262 in AWBRMetXyn5) were detected, compared to thermophilic reference XynAS9 (Figure 3).

### 3.5 The 3D Homology Model Structures of Three Bacterial GH10 Xylanases

The homology models of three GH10 xylanases (AWBRMetXyn5, AWBRMetXyn10, and AWBRMetXyn19) were determined by the SWISS-MODEL homology modeling server based on their specific amino acid sequences. The selection of templates for modeling was done by considering factors such as the sequence identity, the global model quality estimate (GMQE) value, and QMEANDisCo global and coverage values. The most suitable template of three xylanases was found as 8B73, chain 1A from *Acetivibrio clariflavus* β-1,4-xylanase of glycoside hydrolase family 10 (AcXyn10A) deposited in the RCSB PDB databank,^74^ having a sequence identity of 52.38–58.29% and a coverage of 99%. The predicted structures were found to have high quality based on two quality parameters: QMEANDisCo and GMQE values (Table 2).

**Table 2:** Homology Model Scores of 3D Predicted Structures of the Three Probable Thermostable Xylanases from the Rumen of the Anatolian Buffalo

**no.**	**protein ID**	**protein name**	**template**	**sequence identity (%)**	**coverage (%)**	**GMQE**	**QMEANDisCo Global**
1	DIANCIHN_13666	AWBRMetXyn5	8B73	52.88	99	0.88	0.85 ± 0.05
2	MLOJOCKJ_01271	AWBRMetXyn10	8B73	52.38	99	0.88	0.84 ± 0.05
3	ELKBKCFI_43865	AWBRMetXyn19	8B73	58.29	98	0.87	0.84 ± 0.05

Several bioinformatics tools were employed to cross-validate AWBRMetXyn5, AWBRMetXyn10, and AWBRMetXyn19. Structural alignment between the predicted models and the templates revealed significant compatibility and similarity in folding patterns, as depicted in Figures S1a–S3a. The Ramachandran plots showed that 90.7–92.4% of residues in the xylanases occupied the most favored locations, with no outliers indicating unfavorable dihedral angles, as shown in Figures S1b–S3b. Furthermore, the QMEAN4 scores of the enzymes fell within the range of −0.51 to −1.39, as illustrated in Figures S1c–S3c, indicating good quality and similarity to native structures.^75^ Assessment by Verify 3D confirmed that 87–89% of residues in all enzyme structures exhibited an averaged 3D–1D score ≥ 0.2, meeting the 80% score requirement, as depicted in Figures S1d–S3d. To evaluate the global quality of the xylanase homology models, *Z*-scores were calculated by using the ProSA online server. The results showed *Z*-scores ranging from −10.7 to −10.97, as shown in Figures S1e–S3e. These *Z*-scores fell within the range of scores observed for native protein structures of similar size, obtained through experimental techniques such as NMR and X-rays.^50^

### 3.6 Structural Assessment of Three GH10 Xylanases

**Figure 4:**
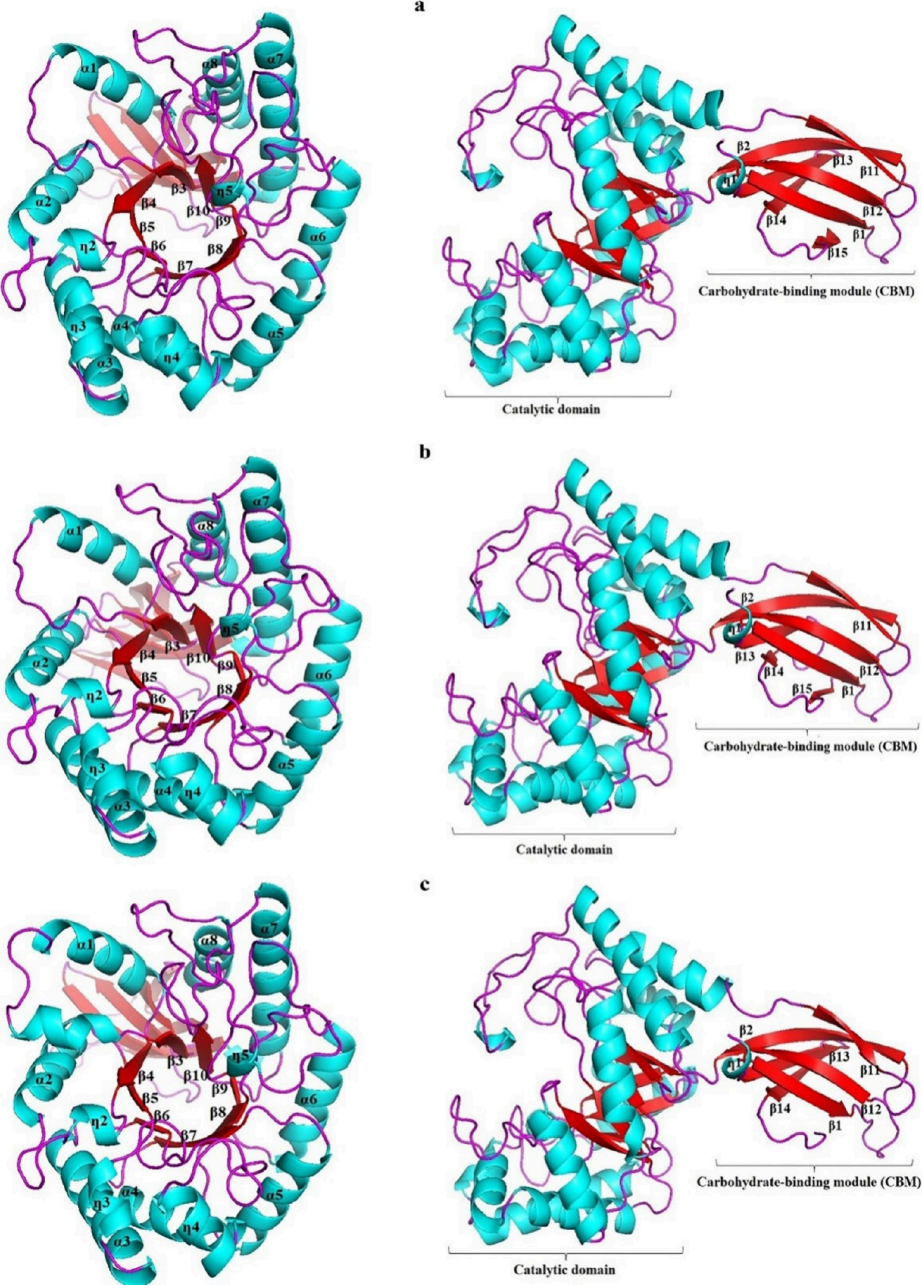
Predicted three-dimensional arrangements of the GH10 xylanases from the rumen of Anatolian water buffalo. (a) AWBRMetXyn5, (b) AWBRMetXyn10, and (c) AWBRMetXyn19.

The present work demonstrated that the domains and their folding patterns were preserved to a significant extent in three predicted models of AWBRMetXyn5, AWBRMetXyn10, and AWBRMetXyn19. Concerning this matter, three xylanases had a TIM-barrel architecture, including eight α-helices and eight β-strands as the catalytic domain. The catalytic residues (two glutamic acid residues E157/E262 in AWBRMetXyn5 and AWBRMetXyn10 and E159/E264 in AWBRMetXyn19) were located on the β6-η4 loop and β9-η5 loop, respectively (Figure 4a–c). In addition, the structural analysis revealed that helix α4 with 15 residues initiated at R180 in AWBRMetXyn5 and AWBRMetXyn10 was longer than that with 13 residues starting at S182 in thermophilic reference xylanase from *Streptomyces* sp*.* S9 (XynAS9) (data not shown).

**Figure 5:**
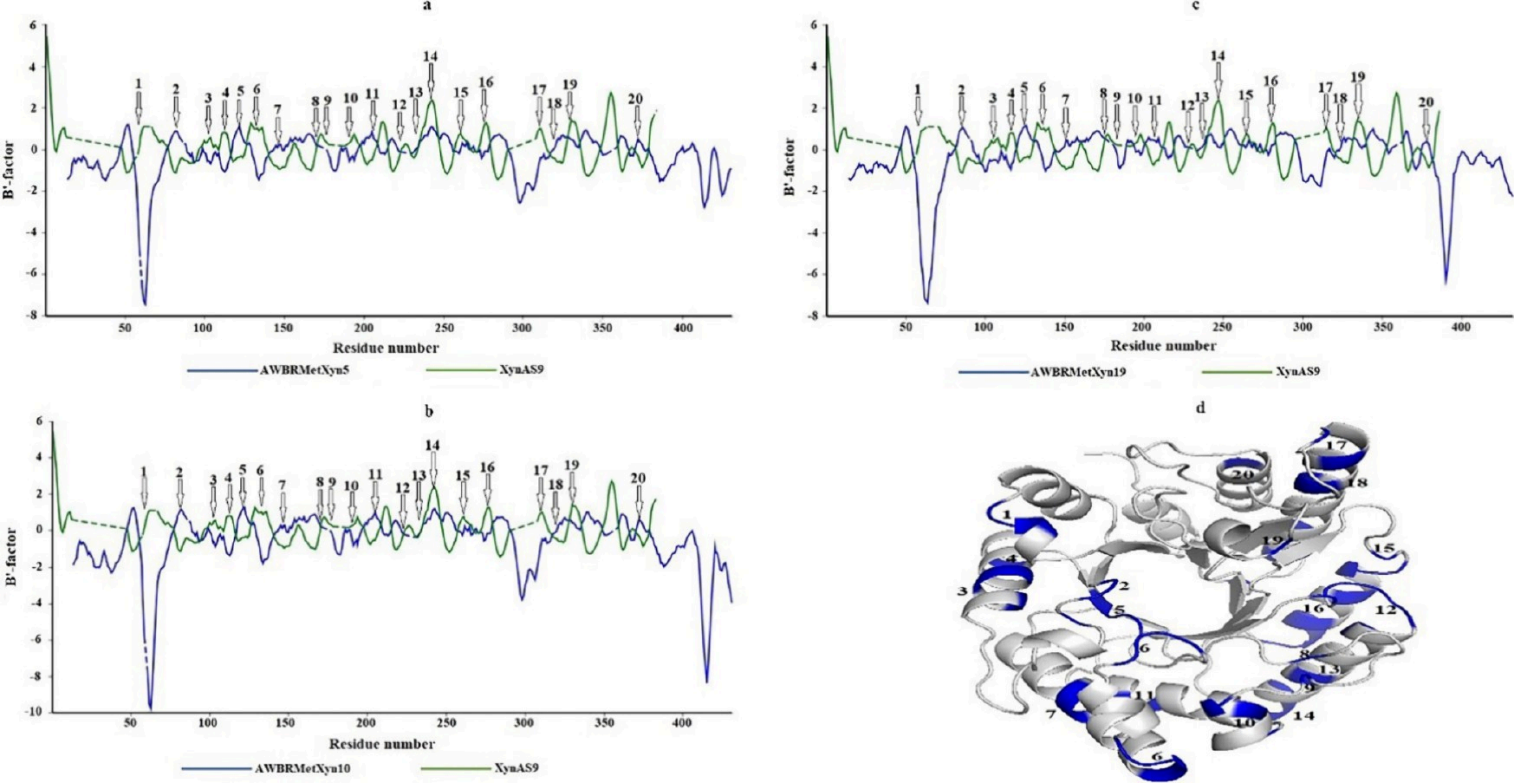
*B*′-factor distribution of the three GH10 xylanases from the rumen of the Anatolian water buffalo, compared to thermophilic reference XynAS9. (a) Alignment of the *B*′-factor profile of AWBRMetXyn5 with the XynAS9, (b) alignment of the *B*′-factor profile of AWBRMetXyn10 with the XynAS9, (c) alignment of the *B*′-factor profile of AWBRMetXyn19 with the XynAS9, and (d) important regions of XynAS9 for thermostability.

Based on the BANΔIT analysis of this work, the *B*′-factor profiles of AWBRMetXyn5, AWBRMetXyn10, and AWBRMetXyn19 were similar to each other and the three enzymes displayed lower *B*′-factor values in a greater number of regions, compared to thermophilic reference XynAS9 (Figure 5a–d). These hotspot regions were adapted to the *B*′-factor distribution of XynAS9 and each GH10 xylanase. According to this result, *B*′-factors (regions 1, 3, 4, 6, 9, 10, 14, 15, 16, 17, and 19) in 11 regions of three xylanases were lower, compared to XynAS9, whereas six regions (regions 2, 5, 7, 11, 18, and 20) of XynAS9 possessed lower *B*′-factor values than each of three xylanases. Region 1 in the N-terminal region of AWBRMetXyn5 (Y52 and E53 on the β3-α1 loop), AWBRMetXyn10 (Y52 on the β3-α1 loop), and AWBRMetXyn19 (L48, F49, and S53 on the β3-α1 short-helix and loop) exhibited noticeable differences, with the *B*′-factor value of the three xylanases being significantly lower when compared to thermophilic reference XynAS9. In contrast, G79 in the α2-helix, M123 in the η3-helix, D150 in the β6-strand, T199 in the β7-strand, A329 in the β7-α2 loop, and G377 in the β13-strand of AWBRMetXyn5 were found in six regions with low *B*′-factor values of XynAS9 (regions 2, 5, 7, 11, 18, and 20). Their corresponding residues were identical in AWBRMetXyn10 and AWBRMetXyn19; however, the aligned residues in XynAS9 were Gln, Glu, Ile, Lys, Ser, and Val, respectively.

In this work, AWBRMetXyn5, AWBRMetXyn10, and AWBRMetXyn19 had a higher number of salt bridges and hydrophobic interactions compared with thermophilic reference XynAS9. Also, each xylanase possessed five short helices (Table 3). In addition, thermostability predictor analysis showed that *T*_m_ scores of model structures of three xylanases were much higher than those of XynAS9 (Table 3).

**Table 3:** *T*_m_ Scores and the Counts of Salt Bridges, Hydrophobic Interactions, and Short Helices of the Three Probable Thermostable Xylanase Models from the Rumen of the Anatolian Water Buffalo, Compared to the XynAS9

**no.**	**protein ID**	**protein name**	**salt bridge**	**hydrophobic interactions**	**short helices**	*T*_**m**_**score (°C)**
1	DIANCIHN_13666	AWBRMetXyn5	50	170	5	70.06
2	MLOJOCKJ_01271	AWBRMetXyn10	51	168	5	74.61
3	ELKBKCFI_43865	AWBRMetXyn19	61	166	5	71.72
4	B4XVN1	XynAS9	43	145	5	53.41

### 3.7 The Interactions Occurring between Enzymes and Substrates

Molecular docking analysis was carried out on AWBRMetXyn5, AWBRMetXyn10, AWBRMetXyn19, and thermophilic reference XynAS9, with 26 different xyloligosaccharides as the ligands. AWBRMetXyn5, AWBRMetXyn10, and AWBRMetXyn19 shared a similar binding affinity to each ligand. Enzyme-ligand docked complexes of three xylanases mostly possessed lower binding energy than XynAS9-ligand complexes, indicating that the three enzymes had a higher binding affinity to the substrates. In addition, three xylanases had the highest binding affinity to the X_4_ ligand, a range of free energy of −12 to −12.4 kCal/mol, whereas the XynAS9-X_4_ docked complex possessed −10.5 kCal/mol (Table S5).

**Figure 6:**
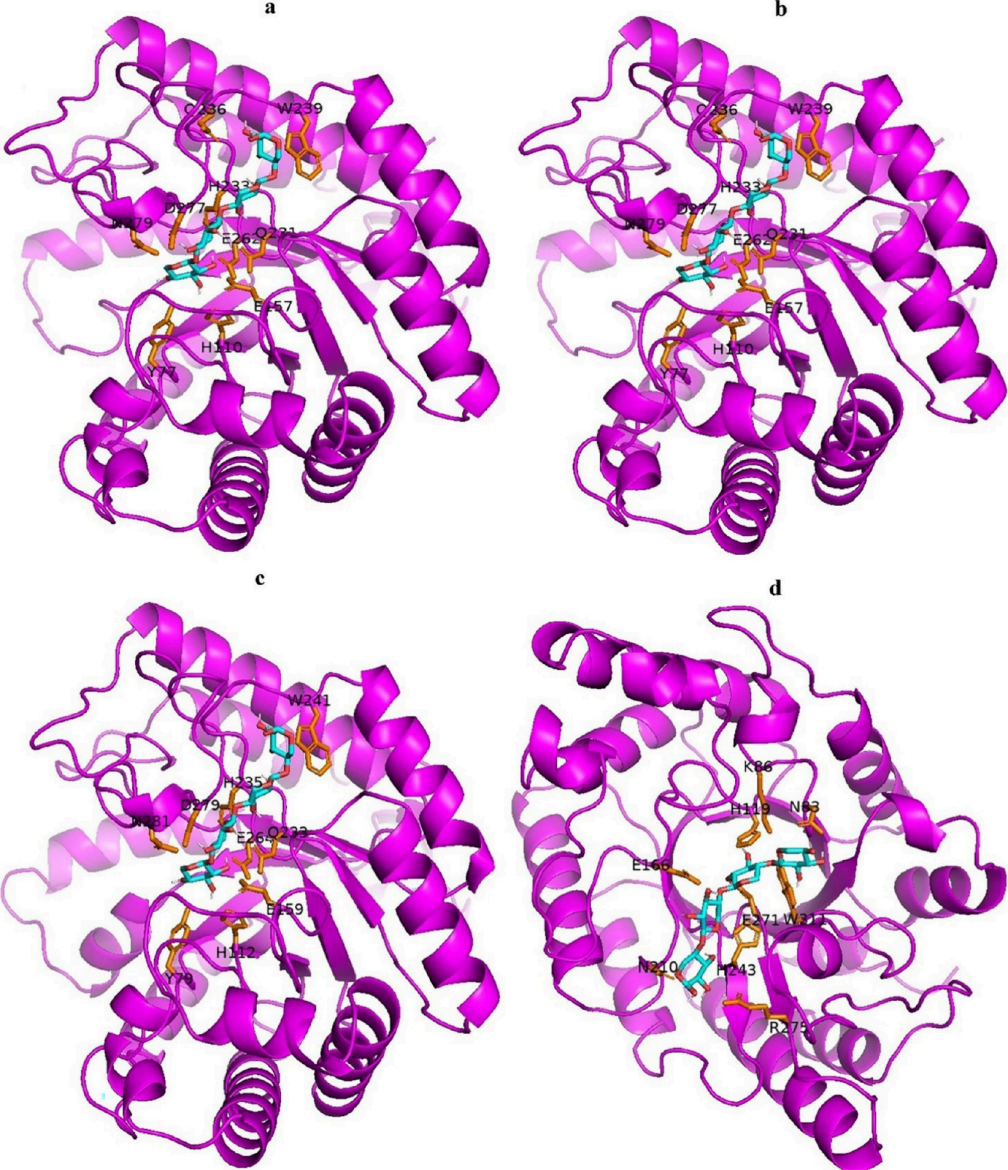
Polar interactions between each of the three xylanases and the X_4_, compared to the XynAS9. (a) AWBRMetXyn5-X_4_, (b) AWBRMetXyn10-X_4_, (c) AWBRMetXyn19-X_4_, and (d) XynAS9-X_4_. The orange color referred to the residues that have hydrogen bonds with the X_4_.

The xylotetraose (X_4_) interactions across three xylanases, relative to XynAS9, are shown in Figure 6. Accordingly, the same residues in three xylanases interacted with the X_4_ ligand, which were corresponding amino acids (Y77, H110, E157, Q231, H233, Q236, W239, E262, D277, and N279 in AWBRMetXyn5) of the enzymes. On the other hand, the residues involved in the ligand interaction in XynAS9 were N83, K86, H119, E166, N210, H243, E271, R275, and W311. Among these residues, the corresponding amino acids K86, H119, E166, H243, and E271 in three xylanases commonly form H bonds with the ligand.

### 3.8 MD Simulation

**Figure 7:**
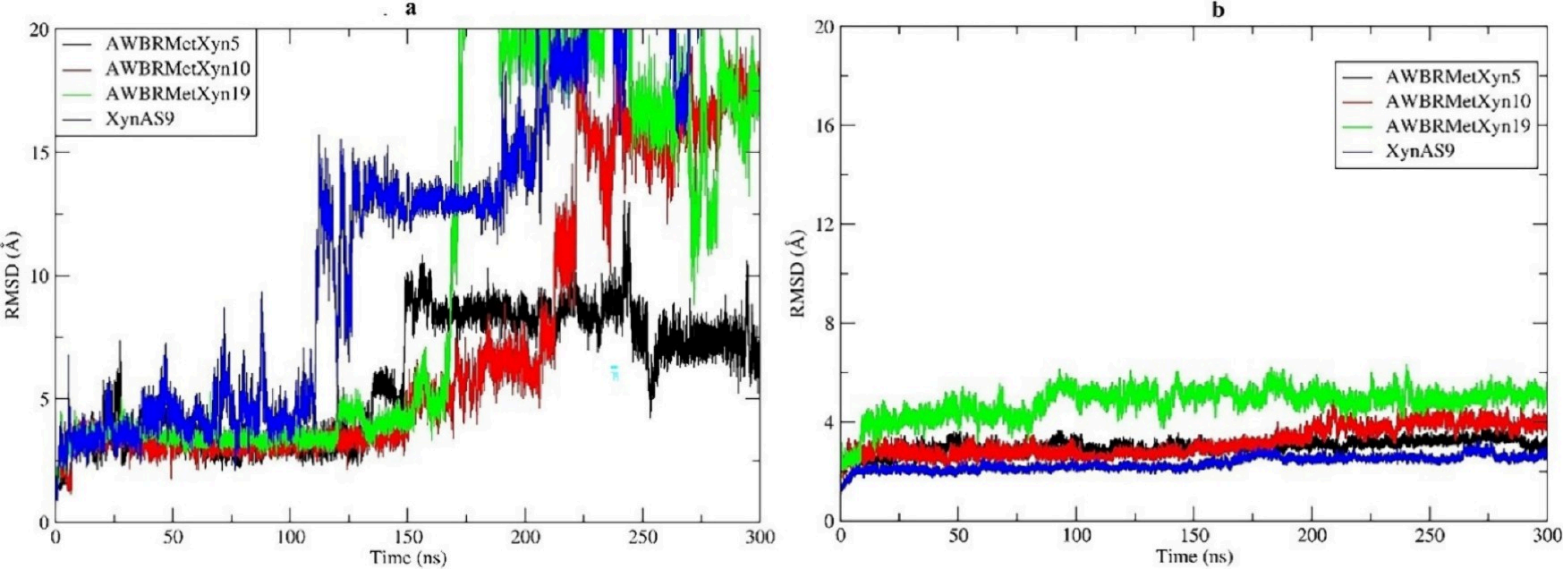
Root-mean-square deviation (RMSD) of three GH10 xylanases relative to XynAS9. (a) Enzyme-X_4_ complex. (b) Enzyme.

MD simulation is a reliable approach for linking protein structure to stability, showing strong agreement with relevant experimental data.^76,77^ In this study, MD simulations were employed to identify key residues interacting with the X_4_ ligand, which exhibited the lowest binding energy among all docked complexes of AWBRMetXyn5, AWBRMetXyn10, and AWBRMetXyn19 in molecular docking analysis, over 300 ns. As a result, the RMSD plot showed that the binding stability of the enzymes varied throughout the simulation time. The AWBRMetXyn5-X_4_ docked complex showed a consistently stable RMSD value throughout the simulation, even during 100–200 ns at increasing temperatures from 300 to 339 K and 200–300 ns at 339 K. In addition, the AWBRMetXyn10-X_4_ docked complex exhibited a stable RMSD value until 200 ns; then, it was seen to fluctuate above 15 Å after 225 ns at 339 K. Also, the AWBRMetXyn19-X_4_ docked complex had a stable RMSD in the first 175 ns; then, the complex fluctuated beyond 20 Å at 175–225 ns; finally, RMSD stayed at 15–20 Å at 225–300 ns. As for the XynAS9-X_4_ docked complex, after about 110 ns, which began to increase temperature toward 339 K, the RMSD value sharply fluctuated to a 15 Å distance, where it stayed stable until 200 ns, and then, it reached a 20 Å fluctuation up to the end of the simulation. Thus, AWBRMetXyn5-X_4_ and relatively AWBRMetXyn10-X_4_ docked complexes appeared to have a more stable RMSD value over time simulation, compared to the AWBRMetXyn19 and thermophilic reference XynAS9 (Figure 7a). In addition, the RMSD graph of each enzyme without a ligand was created along the 300 ns simulation. In addition, this analysis showed that three xylanases and the reference enzyme XynAS9 had a similar stable RMSD profile along the simulation (Figure 7b).

**Figure 8:**
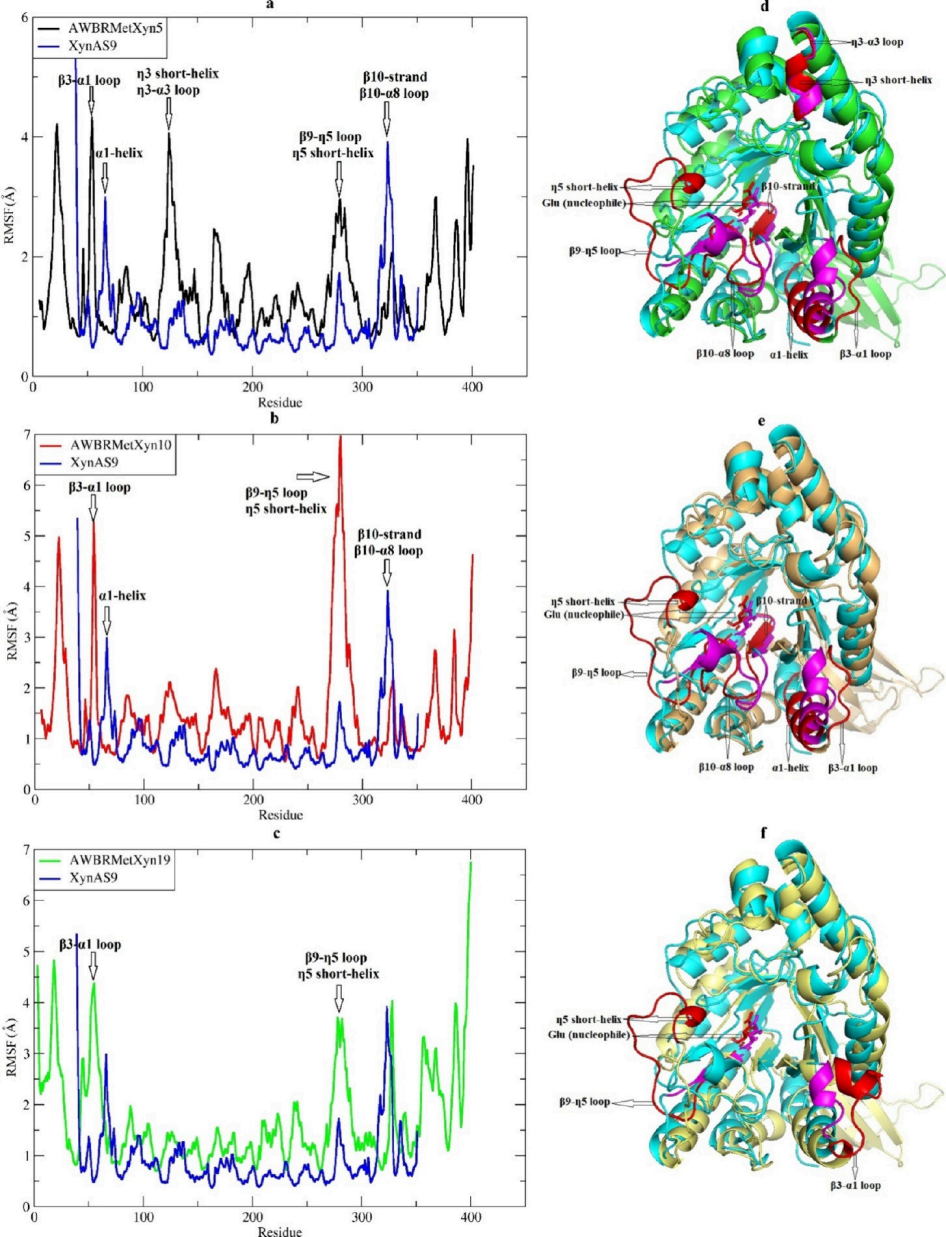
Root-mean-square fluctuation (RMSF) and superimposed structures of three xylanases with XynAS9. (a) RMSF graph of AWBRMetXyn5, (b) RMSF graph of AWBRMetXyn10, (c) RMSF graph of AWBRMetXyn19, (d) superimposed structure of AWBRMetXyn5, (e) superimposed structure of AWBRMetXyn10, and (f) superimposed structure of AWBRMetXyn19. The blue line and cyan color represented the RMSF values and the structure of XynAS9, respectively.

The RMSF values for AWBRMetXyn5, AWBRMetXyn10, AWBRMetXyn19, and thermophilic reference XynAS9 were calculated during the MD simulation by evaluating the fluctuation of each residue’s position to its average structure. It was determined that AWBRMetXyn5, AWBRMetXyn10, and AWBRMetXyn19 showed greater fluctuations in amino acid movements in three common regions relative to the XynAS9: the β3-α1 loop, β9-η5 loop, and η5 short-helix. AWBRMetXyn5 and AWBRMetXyn10 possessed a β3-α1 loop, whereas XynAS9 contained a short-helix and AWBRMetXyn19 had a loop along with a short-helix, in the aligned region of the β3-α1 loop. In addition, AWBRMetXyn5 and AWBRMetXyn10 displayed three regions with lower fluctuations compared to XynAS9 and AWBRMetXyn19: the α1-helix, β10-strand, and β10-α8 loop. Additionally, AWBRMetXyn5 had a greater fluctuation in two regions compared to XynAS9, AWBRMetXyn10, and AWBRMetXyn19: the η3 short-helix and the η3-α3 loop (Figure 8a–f).

**Figure 9:**
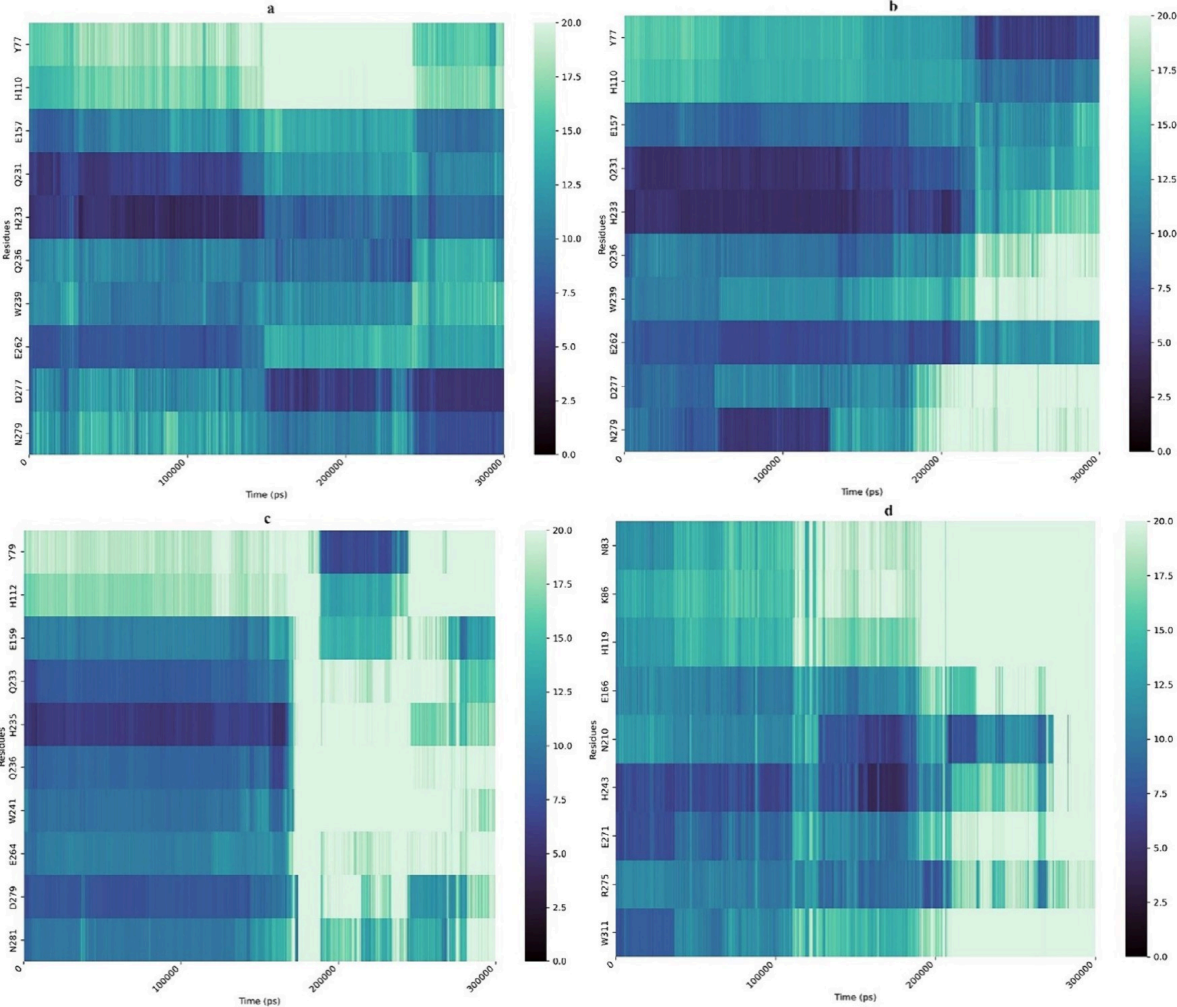
Average distance analysis of MD simulation of three thermostable xylanases with X_4_ (a) AWBRMetXyn5, (b) AWBRMetXyn10, (c) AWBRMetXyn19, and (d) XynAS9. Heat maps’ distance (Angstrom) color distribution.

The dynamics analysis of the average distance was conducted for three GH10 enzymes and the thermophilic reference XynAS9 to measure the distance between the center of mass (COM) of the ligand and the COM of each binding pocket residue identified in the docking results. The results were visualized as a heat map. This analysis showed that the ligand-interacted residues in AWBRMetXyn5 and AWBRMetXyn10 were relatively closer to the ligand compared to AWBRMetXyn19 and XynAS9. Regarding this, most of the residues (Q231, H233, Q236, W239, D277, and N279) except Y77 and H110 in AWBRMetXyn5 remained in similar distances (2.5–12.5 Å) to the ligand along the 300 ns (Figure 9a). In AWBRMetXyn10, Y77, H110, Q231, and H233 maintained their distance to the ligand, fluctuating between 5 and 15 Å, while the remaining residues (Q236, W239, D277, and N279) moved farther away from the ligand, exceeding 20 Å, particularly during the 200–300 ns interval (Figure 9b). In the case of the other enzymes (AWBRMetXyn19 and XynAS9), the distance between the corresponding residues and the ligand exceeded 20 Å during the 200–300 ns interval at 339 K, despite remaining stable up to approximately 180–200 ns. Similar results were obtained for the other residues of AWBRMetXyn19 and XynAS9 (Figure 9c,d).

## 4 Discussion

In this study, rumen metagenomes of three Anatolian water buffalos were sequenced to determine the microbial diversity and xylanase sequences, and three GH10 xylanases were *in silico* characterized at the sequence and structure level, relative to thermophilic reference xylanase from *Streptomyces* sp*.* S9 (XynAS9). *In silico* analyses are solely computational analyses and should be validated with experimental findings. This study, which consists of *in silico* analyses, has been discussed by comparing it with the previously characterized thermophilic xylanases to strengthen its findings.

Many studies have examined rumen microbial composition in various ruminants (e.g., Angus bulls, Hu lambs, Holstein calves, and Hainan black goat kids) under different dietary supplements, along with the production of lignocellulose-degrading enzymes like xylanases. These studies have associated the abundance of *Ruminococcus flavefaciens*, *Prevotella ruminicola*, *Ruminococcus albus*, *Fibrobacter succinogenes*, *Butyrivibrio fibrisolvens*, and *Ruminobacter amylophilus* with the production of lignocellulose-degrading enzymes including xylanases.^78−83^ For example, recent work has indicated that the rumen in the dry roughage-fed buffalo possessed a greater number of bacterial genera including *Fibrobacter*, *Clostridium*, *Prevotella*, and *Ruminococcus*, which play a role in breaking down plant biomass.^32^ In this work, it was revealed that *Prevotella*, *Butyrivibrio*, *Ruminococcus*, *Fibrobacter*, and *Clostridium* were found as dominant genera in three rumen samples (Figure 1b). These genera were part of higher-level hierarchical groups: *Clostridium* (Clostridiales bacterial order), *Ruminococcus* (Oscillospiraceae bacterial family), *Prevotella* (Bacteroidales bacterial order), and *Butyrivibrio* (Lachnospiraceae bacterial family). Supporting this, Blastp analysis of this work showed that the majority of 19 full-length xylanase sequences from three rumens belonged to Clostridiales bacterial order, Oscillospiraceae bacterial family, Bacteroidales bacterial order, and Lachnospiraceae bacterial family (Table S3). In line with this, a recent study showed that lignocellulosic substrates enrich ruminal microbiota with hydrolytic bacteria, mainly from the Clostridiales order, Lachnospiraceae family, and Bacteroidales order. The same study has also indicated that cellulase and xylanase enzymes were released in significant amounts when these bacteria were coinoculated.^84^ In another work, Deng et al. have indicated that the primary degraders of polysaccharides in the ruminal microbiota were the members of the Clostridiales order.^80^ Thus, this study strongly suggested that obtaining xylanase enzyme sequences probably belonged to the genera *Clostridium, Ruminococcus*, *Prevotella*, and *Butyrivibrio*.

Biophysicochemical features of the enzyme sequences, such as the instability index, aliphatic index, and theoretical *T*_m_ value, are significant to understand the nature of the enzymes. The instability index of a sequence below 40 indicated a stable protein structure, and a higher aliphatic index and *T*_m_ referred to higher thermal stability.^85^ In this study, the biophysicochemical analysis showed that AWBRMetXyn5, AWBRMetXyn10, and AWBRMetXyn19, among other full-length xylanases, had a higher *T*_m_ value (above 65 °C), a relatively high aliphatic index (above 80), and a low instability index (in a range of 31–39) (Table 1), comparable with the thermostable xylanases in the literature. Accordingly, a work has demonstrated that bacterial GH10 xylanase from *Streptomyces* sp*.* S9 had an optimum temperature of 60 °C and exhibited high thermal stability retaining its activity over 65% at 80 °C.^86^ In this study, its aliphatic index and instability index were found as 73.64 and 30.01, respectively (Table S4). Another study has shown that a bacterial thermostable alkaline active endo-β-1,4-xylanase from *Bacillus halodurans* S7, which had an aliphatic index of 78.31 and an instability index of 32.69 (Table S4), possessed a high optimum temperature of 70–75 °C.^87^ The optimum temperature of xylanase from bacterial thermoalkaline *Anoxybacillus* sp*.* E2 was found as 65 °C,^88^ and its aliphatic and instability indexes were determined as 85.27 and 38.93, respectively (Table S4). In addition, fungal GH11 thermostable xylanase from *Thermomyces lanuginosus* possessed an optimum temperature of 65 °C. This enzyme is widely used in industrial applications and retains its 94% activity even after 24 h at 65 °C.^89^ In this work, the enzyme had instability and aliphatic indexes of 26.14 and 62.04, respectively (Table S4). Thus, this work proposed that AWBRMetXyn5, AWBRMetXyn10, and AWBRMetXyn19 might possess highly thermostable characters.

The molecular phylogeny analysis revealed that AWBRMetXyn5, AWBRMetXyn10, and AWBRMetXyn19 were clustered within the GH10 family, with the closest homologues of xylanases from different thermophilic bacteria, including *Acetivibrio thermocellus* (UniProt ID A3DIL1),^90,91^*Acidothermus cellulolyticus* (UniProt ID A0LRT6),^92^*Anoxybacillus* sp*.* E2 (UniProt ID D7NNK8),^88^ and *Streptomyces lividans* (UniProt ID P26514)^93^ (Figure 2). In addition, the most suitable template of homology models of three xylanases was determined as xylanase from *Acetivibrio clariflavus* (Table 2). *Acetivibrio clariflavus*, originally known as *Clostridium clariflavum*,^94^ is a thermophilic and anaerobic bacterium.^95−97^ Its environmental isolate optimally grows at 60 °C, and it is a sole thermophilic microorganism growing on xylose, xylooligomers, or other hemicellulose components^98^ and efficiently degrades the hemicellulose and xylan.^99^ Even though the xylanase from *Acetivibrio clariflavus* has not been biochemically characterized, it is well-known that this bacterium is a source of thermostable enzymes. Regarding this, the recombinant pure xylosidase enzyme exhibits stability at temperatures as high as 90 °C for 4 h, maintaining approximately 54.6% of its relative activity in comparison to the control.^100^ In another recent work, the purified recombinant cellobiohydrolase enzyme remained stable and displayed residual activities of 63% when subjected to a 1 h incubation at 80 °C, in comparison to the control.^101^ Taken together, the association of thermophilic xylanases within closely related phylogenetic clusters, along with the fact that the xylanase used as a template in homology modeling originates from a thermophilic bacterium, strongly suggests that AWBRMetXyn5, AWBRMetXyn10, and AWBRMetXyn19 are likely to function effectively at high temperatures.

Many conserved residues including two catalytic amino acids were detected compared to the XynAS9. Based on the well-known GH10 catalytic mechanism, the active center of thermophilic reference XynAS9 contains two conserved catalytic glutamate residues, E166 and E271. These residues have specific roles within the catalytic mechanism, with E166 serving as the general acid/base and E271 acting as the nucleophile.^102^ In addition, Chen et al. have shown that 12 residues (N83, K86, H119, W123, N165, E166, Y209, Q241, E271, R275, W311, and W319) in XynAS9 highlighted their crucial roles in interacting with substrates and they exhibited either strict conservation or semiconservation in the GH10 family.^103^ The alignment analysis revealed that the corresponding eight residues (H119, W123, N165, E166, Q241, E271, W311, and W319) were conserved in three xylanases (AWBRMetXyn5, AWBRMetXyn10, and AWBRMetXyn19) (Figure 3).

The GH10 family members possess a distinctive elliptical (β/α)_8_-barrel, also known as the TIM-barrel, architecture. This structure consists of eight β-strands forming the central barrel and eight α-helices forming the outer layer.^104^ This structural characteristic is common to various glycoside hydrolase (GH) families, such as GH10 and GH11 xylanases, as well as glycosidase esterases.^8,105^ In this work, the structural assessment indicated that the domains and their folding patterns of AWBRMetXyn5, AWBRMetXyn10, and AWBRMetXyn19 were highly similar to those of GH10 xylanases. Three xylanases had a catalytic domain with a TIM-barrel folding pattern and carbohydrate-binding module (CBM) (Figure 4). A protein engineering work on XynAS9 showed that five mutants including D185P/S186E and V81P/G82E/D185P/S186E possessed higher thermal performance than XynAS9 and increased the melting temperature of the enzyme by 2.3 and 11.2 °C, respectively.^106^ In this work, alignment analysis showed that corresponding residues of V81, G82, D185, and S186 were Y71, T72, V181, and E182 in two xylanases (AWBRMetXyn5 and AWBRMetXyn10) (Figure 3). The structural analysis revealed that α4-helices including V181 and E182 (initiating at R180) in two xylanases were longer than that (starting at S186) in XynAS9 with the corresponding residues D185 and S186 (data not shown). D185 and its neighbor G184 were a part of the η4-α4 loop in XynAS9, whereas the corresponding residues (R180 and V181) in two xylanases were a part of the α4-helix. The substitution of S186E, which was a substitution in D185P/S186E and V81P/G82E/D185P/S186E mutants exhibiting higher thermal performance,^106^ might make a longer α-helix in AWBRMetXyn5 and AWBRMetXyn10 and become more thermostable than XynAS9.

BANΔIT analysis showed that all three xylanases (AWBRMetXyn5, AWBRMetXyn10, and AWBRMetXyn19) displayed lower *B*′-factor values of the β3-α1 loop/short-helix in the N-terminal site, compared to thermostable reference xylanase (XynAS9) (Figure 5a–d), which is a GH10 family member elaborately characterized at biochemical, sequence, and structure levels.^86,103^ In a work, it has been suggested that the N-terminal coil of the Xyn10A_ASPNG, from *Aspergillus niger*, is significant for the stabilization of the GH10 xylanase structure.^107^ In line with this, replacing the N-terminal peptide of the mesophilic xylanase AoXyn11A with the N-terminal peptide of the thermophilic protein EvXyn11TS resulted in a significant enhancement in the stability of the recombinant protein, with an increase of approximately 200-fold.^108^ In addition, two studies have emphasized that the N-terminal region of bacterial and fungal xylanases is important for the thermostability of the xylanases.^109−111^ Thus, this study suggested that the β3-α1 loop/short-helix may play a role in the thermostability of AWBRMetXyn5, AWBRMetXyn10, and AWBRMetXyn19. In addition, this analysis showed that the residues in the regions having higher *B*′-factor values of AWBRMetXyn5 were G79, M123, D150, T199, A329, and G377, and the aligned amino acids were the same in AWBRMetXyn10 and AWBRMetXyn19, compared to the XynAS9 including Gln, Glu, Ile, Lys, Ser, and Val, respectively, in those regions (Figure 5a–d). Among these, the substitutions (M123E and T199K) can potentially provide the formation of new salt bridges with another basic and acidic residue, respectively, whereas D150I and G377V can form hydrophobic interactions with other hydrophobic residues. G79Q and A329S can potentially form an extra polar interaction with (an)other polar residues. Thus, this work suggested that G79Q, M123E, D150I, T199K, A329S, and G377V might be potential targets to improve the thermostability of three enzymes.

Noncovalent interaction analysis showed that three xylanases (AWBRMetXyn5, AWBRMetXyn10, and AWBRMetXyn19) exhibited more salt bridges and hydrophobic interactions than XynAS9, while all three contained five short helices, similar to those of XynAS9 (Table 3). De Souza et al. have shown that the increase in thermostability of XynA may be attributed to a greater occurrence of weak interactions and structural abnormalities (such as salt bridges, hydrophobic interactions, and shorter helices) compared to its native form.^112^ This knowledge is supported by other works in the literature.^113−118^ In addition, thermostability predictor analysis demonstrated that three xylanases possessed a much higher *T*_m_ score than XynAS9 (Table 3).

MD simulation revealed that the three xylanases (AWBRMetXyn5, AWBRMetXyn10, and AWBRMetXyn19) exhibited larger fluctuations in amino acid movements of the β3-α1 loop in the RMSF plot compared to XynAS9 (Figure 8a–f). Accordingly, AWBRMetXyn5 and AWBRMetXyn10 featured the same long loop (β3-α1 loop) of 10 amino acids in this region (Figure 8d,e), and AWBRMetXyn19 (Figure 8f) had a 15-amino-acid loop along with a short-helix in the corresponding site; however, XynAS9 contained a short-helix consisting of 4 amino acids and a loop of 2 amino acids located at the aligned region of the β3-α1 loop. A common characteristic of the aligned region between the β3-strand and α1-helix in the three xylanases was that it was longer compared to XynAS9. BANΔIT analysis indicated that the β3-α1 loop in the N-terminal site of AWBRMetXyn5 and AWBRMetXyn10 had a lower *B*′-factor value than XynAS9 (Figure 5), suggesting that it might contribute to their thermostability. The β3-α1 loop may have contributed to the formation of a greater number of noncovalent interactions in AWBRMetXyn5, AWBRMetXyn10, and AWBRMetXyn19 during MD simulation compared with XynAS9, thereby potentially making a positive contribution to their thermostability. Similarly, the β9-η5 loop in three xylanases had more fluctuation and was longer than the aligned region in XynAS9. The β9-η5 loop had catalytic nucleophile glutamic acid (Figure 3). A greater fluctuation in the β9-η5 loop in the three xylanases may enhance the interaction of the nucleophile with the ligand. Indeed, distance analysis showed that the nucleophiles in particularly AWBRMetXyn5 and AWBRMetXyn10 remained at closer distances to the ligand throughout the simulation (Figure 9a–d). Therefore, this study suggested that AWBRMetXyn5 and AWBRMetXyn10 may exhibit higher activity at elevated temperatures compared to XynAS9 and AWBRMetXyn19. Additionally, AWBRMetXyn5 exhibited higher fluctuation in two regions (η3 short-helix and η3-α3 loop) different from AWBRMetXyn10 and AWBRMetXyn19, compared to XynAS9 (Figure 8a–c). Indeed, AWBRMetXyn5 and AWBRMetXyn10 had highly similar amino acid sequences by 98.76% identity, and they globally possessed the differences in five amino acids (I256, N345, T392, N396, and T397 for AWBRMetXyn5; L256, D345, S392, K396, and A397 for AWBRMetXyn10) (Figure 3). Among these differences, three residues in AWBRMetXyn5 (N345, N396, and T397) had different properties from corresponding residues in AWBRMetXyn10. Two of these (N396 and T397) were found in their carbohydrate-binding module. They may have led to the difference in substrate binding of the enzymes. Distance analysis revealed that AWBRMetXyn5 had a greater number of ligand-binding residues positioned closer to the ligand than AWBRMetXyn10, suggesting that AWBRMetXyn5 had a stronger binding affinity.

*In silico* studies have some limitations due to the lack of biological context in predictions, oversimplification of complex biological systems, and the possibility of biased evaluations due to the limited number of characterized enzymes in databases and the literature.^119−121^ However, *in silico* analyses at the sequence, structural, and dynamic levels of enzymes have been successful in selecting the best enzymes from a large number^122^ or in supporting experimental findings.^123^ Such analyses provide promising findings for protein engineering, both in selecting the enzymes with the best properties and in targeting specific amino acids and regional differences in enzymes.^110,124,125^ Therefore, in this study, enzymes were evaluated using different methods at the sequence, structure, and dynamic levels, and some cross-validations were performed by using computational analyses. Nevertheless, the key findings obtained in this study still require experimental validation. For this reason, the experimental validation of three enzymes (AWBRMetXyn5, AWBRMetXyn10, and AWBRMetXyn19) and their characterization will be performed in a future study. Additionally, state-of-the-art protein engineering strategies such as the combinatorial active site test (CAST), iterative saturation mutagenesis (ISM), or site saturation mutagenesis (SSM)^126^ could be employed to target specific residues in the β3-α1 loop, β9-η5 loop, η3 short-helix, η3-α3 loop, and three residues (N345, N396, and T397) in AWBRMetXyn5. In parallel with this, site-directed mutagenesis would be a suitable approach for substituting specific residues (G79Q, M123E, D150I, T199K, A329S, and G377V) in AWBRMetXyn5.

## 5 Conclusions

The present work had shotgun sequencing of rumen metagenomes of three Anatolian water buffalos, the investigation of the relationship between microbial flora and xylanases, and *in silico* analyses of the thermostable xylanases to consider at the sequence, structure, and dynamics level. In this work, shotgun sequence and Blastp analyses showed that *Clostridium* (Clostridiales bacterial order), *Ruminococcus* (Oscillospiraceae bacterial family), *Prevotella* (Bacteroidales bacterial order), and *Butyrivibrio* (Lachnospiraceae bacterial family) were found as dominant potential xylanase-producer genera in three rumen samples. Furthermore, the biophysicochemical analysis indicated that three xylanases (AWBRMetXyn5, AWBRMetXyn10, and AWBRMetXyn19) exhibited an aliphatic index above 80, an instability index below 40, and melting temperatures (*T*_m_) surpassing 65 °C. These characteristics suggested that they may have a greater thermophilic tendency than the other xylanases. Phylogenetic analysis classified AWBRMetXyn5, AWBRMetXyn10, and AWBRMetXyn19 into the GH10 family, grouping them with thermophilic xylanases, and homology modeling identified the best template as a xylanase from a thermophilic bacterium. BANΔIT analysis showed that AWBRMetXyn5, AWBRMetXyn10, and AWBRMetXyn19 possessed lower *B*′-factor values in a β3-α1 loop/short-helix in the N-terminal site compared to reference thermophilic XynAS9, suggesting that this region might contribute to their thermostability. Furthermore, this analysis revealed that the residues in the regions with higher *B*′-factor values in AWBRMetXyn5 were G79, M123, D150, T199, A329, and G377. The aligned amino acids in AWBRMetXyn10 and AWBRMetXyn19 were identical with those in AWBRMetXyn5, whereas XynAS9 contained Gln, Glu, Ile, Lys, Ser, and Val at these positions, respectively. Therefore, this study suggested that the amino acid substitutions (G79Q, M123E, D150I, T199K, A329S, and G377V) could serve as potential replacements for enhancing the thermostability of the three enzymes. RMSF plot analysis of MD simulation indicated that the β9-η5 loop including catalytic nucleophile glutamic acid in three xylanases had more fluctuation and was longer than the aligned region in XynAS9. A greater fluctuation in the β9-η5 loop in three xylanases may enhance the interaction of the nucleophile with the ligand. The distance analysis of MD simulation indicated that the nucleophiles in particular AWBRMetXyn5 and AWBRMetXyn10 remained at closer distances to the ligand throughout the simulation, compared to XynAS9 and AWBRMetXyn19, supporting the RMSF analysis results. Additionally, according to the MD simulation analysis results, the most striking difference in AWBRMetXyn5 compared to AWBRMetXyn10 was the presence of greater amino acid fluctuations in two distinct regions: the η3 short-helix and the η3-α3 loop. Besides this, AWBRMetXyn5 had a higher number of ligand-binding amino acids positioned closer to the ligand compared to AWBRMetXyn10, whereas AWBRMetXyn5 and AWBRMetXyn10 globally shared a sequence identity of 98.76%. This difference was thought to be related to three key amino acid positions between the two enzymes (N345, N396, and T397 in AWBRMetXyn5; D345, K396, and A397 in AWBRMetXyn10). Taken together, AWBRMetXyn5 could be regarded as a promising candidate for various high-temperature industrial applications, including biofuel production, animal feed, paper pulp manufacturing, and food industry.

This research may also guide efforts to improve the thermostability and catalytic efficiency, key industrial traits of xylanases through rational and/or semirational protein engineering strategies. Specific amino acid substitutions (G79Q, M123E, D150I, T199K, A329S, and G377V) and residues in the β3-α1 loop of AWBRMetXyn5 would be good targets for the enhancement of the thermostability. Additionally, improving catalytic efficiency would involve targeting specific residues (N345, N396, and T397) along with amino acids in the β9-η5 loop, the η3 short-helix, and the η3-α3 loop of AWBRMetXyn5. The key findings obtained from this work should be experimentally validated. Thus, AWBRMetXyn5, AWBRMetXyn10, and AWBRMetXyn19 will undergo experimental characterization and be evaluated for potential industrial applications, and the above-mentioned targets will be tested for AWBRMetXyn5 by applying protein engineering strategies.

## Supporting Information Available

Sequencing statistics; xylanolytic, cellulolytic, and pectinolytic genes; BLAST; biophysicochemical properties; molecular docking; cross-validation of enzyme models (PDF)
