# Supplementary material for: Discovery and In Silico Characterization of Anatolian Water Buffalo Rumen-Derived Bacterial Thermostable Xylanases: A Sequence-Based Metagenomic Approach
Source: ACS Omega. 2025 Mar 18;10(12):12679–98. doi: 10.1021/acsomega.5c00965 (PMC11966585; doi:10.1021/acsomega.5c00965)
Supplement: Supplementary file 1 — ao5c00965_si_001.pdf [file ao5c00965_si_001.pdf]

Supporting Information

The discovery and *in silico* characterization of Anatolian water buffaloe rumen-derived bacterial thermostable xylanases: A sequence-based metagenomic approach

Halil Kurt[1], Dilek Sever Kaya[2], İsmail Akçok[3], Ceyhun Sarı[3], Ebru Albayrak[4], Hasan Murat Velioğlu[4], Hasan Ersin Şamlı[5], Mehmet Levent Özdüven[5], Yusuf Sürmeli[4*]

1. Department of Medical Biology, Hamidiye International School of Medicine, University of Health Sciences, 34668, Istanbul, Turkey
2. Clinical Nutrition and Microbiota Research Laboratory, Istanbul Faculty of Medicine, Istanbul University, 34390, İstanbul, Turkey
3. Department of Bioengineering, Faculty of Life and Natural Sciences, Abdullah Gul University, 38080, Kayseri, Turkey
4. Department of Agricultural Biotechnology, Faculty of Agriculture, Tekirdag Namik Kemal University, 59030, Tekirdag, Turkey
5. Department of Animal Science, Faculty of Agriculture, Tekirdag Namik Kemal University, 59030, Tekirdag, Turkey

Corresponding Author

Assoc. Prof. Dr. Yusuf Sürmeli

Department of Agricultural Biotechnology, Faculty of Agriculture, Tekirdag Namik Kemal University, Süleymanpaşa, Tekirdag, Turkey

Phone: +90 2822502249; Fax: +90 2822509929

E-mail: ysurmeli@nku.edu.tr

Table S1. The sequencing statistics of rumen metagenome obtained from three male Anatolian water buffalo (YR1, YR2 and YR3).

**Bioinformatics scores**	**YR1**	**YR2**	**YR3**
Total reads (300 bp)	20740437	21631029	17711914
Reads after trimming (%), >Q30	20529585	21391284	17504498
Total number of contigs	1519260	1512567	1237300
Number of contigs (>1 kb)	83908	63337	78688
Contig N50 (bp)	506	475	520
Contig L50 (bp)	470762	503761	371344
Total bases in contigs (bp)	791374080	743703283	658132506
Total bases in contigs (>1 kb)	140305500	96274743	131127461
Predicted gene	47507	24892	46482
rRNA	45	28	29
tRNA	570	236	609

Table S2. Xylanolytic, cellulolytic and pectinolytic genes obtained from metagenome sequences of Anatolian water buffalo rumen

**Xylanolytic enzymes**	**EC number**	**number**
Endo-1, 4-β-xylanase	3.2.1.8	976
β-xylosidase	3.2.1.37	304
α-L-arabinofuranosidase	3.2.1.55	275
α-D-glucuronidases	3.2.1.1	880
β-Mannanase	3.2.1.78	297
β-Mannosidase	3.2.1.25	115
Acetyl xylan esterase	3.1.1.72	81
Feruloyl esterase	3.1.1.73	13
Glucuronoyl esterase	3.1.1.B11	0
Lytic polysaccharide monooxygenase	N.A.	0
**Total**		**2941**
**Cellulolytic enzymes**	**EC number**	**number**
Endo-cellulase/endoglucanase	3.2.1.4	1238
Exo-cellulase/cellobiohydrolase	3.2.1.91	100
**Total**		**1338**
**Pectinolytic enzymes**	**EC number**	**number**
Endo-polygalacturonase	3.2.1.15	186
Exo-polygalacturonase	3.2.1.67	38
Exo-polygalacturonase	3.2.1.82	10
Pectin methyl esterase	3.1.1.11	174
Pectin acetyl esterase	3.1.1.6, 3.1.1.86	9
Rhamnogalacturonan I rhamnohydrolase	3.2.1.174	3
Rhamnogalacturonan I galacturonohydrolase	3.2.1.173	7
Rhamnogalacturonan I hydrolase	3.2.1.171	11
**Total**		**438**

Table S3. The closest microorganisms and their alignment results (amino acid sequence identity, E-value, and coverage) of 19 full-length bacterial xylanases from the rumen of the Anatolian water buffalo

No	Protein ID	Protein name	Organism	aa sequence identity (%)	E-value	Coverage (%)
1	DIANCIHN_00801	AWBRMetXyn1	*Oscillospiraceae* bacterium	100	0.0	100
2	DIANCIHN_01743	AWBRMetXyn2	*Clostridiales* bacterium	98.60	0.0	100
3	DIANCIHN_02209	AWBRMetXyn3	*Clostridiales* bacterium	87.46	4.47e-225	100
4	DIANCIHN_11883	AWBRMetXyn4	*Clostridiales* bacterium	99.20	1.58e-285	100
5	DIANCIHN_13666	AWBRMetXyn5	*Clostridiales* bacterium	98.76	1.92e-302	100
6	DIANCIHN_33359	AWBRMetXyn6	*Clostridiales* bacterium FE2011	99.20	2.24e-285	100
7	DIANCIHN_39032	AWBRMetXyn7	*Clostridiales* bacterium	90.73	4.57e-284	100
8	DIANCIHN_41040	AWBRMetXyn8	*Clostridiales* bacterium	99.41	0.0	100
9	DIANCIHN_44290	AWBRMetXyn9	*Prevotella* sp.	91.00	7.45e-255	100
10	MLOJOCKJ_01271	AWBRMetXyn10	*Clostridiales* bacterium	98.01	2.24e-301	100
11	MLOJOCKJ_01367	AWBRMetXyn11	*Clostridiales* bacterium	97.56	6.99-304	100
12	MLOJOCKJ_03719	AWBRMetXyn12	*Clostridiales* bacterium	97.77	0.0	100
13	MLOJOCKJ_07132	AWBRMetXyn13	*Bacteroidales* bacterium	90.53	2.29e-249	100
14	MLOJOCKJ_14190	AWBRMetXyn14	*Oscillospiraceae* bacterium	99.22	2.75e-287	100
15	MLOJOCKJ_18013	AWBRMetXyn15	*Oscillospiraceae* bacterium	100	9.27e-291	100
16	MLOJOCKJ_21568	AWBRMetXyn16	*Bacteroidales* bacterium	98.41	0.0	92
17	ELKBKCFI_13369	AWBRMetXyn17	*Anaerolineaceae* bacterium	99.74	8.56e-287	100
18	ELKBKCFI_26736	AWBRMetXyn18	*Lachnospiraceae* bacterium	80.63	0.0	100
19	ELKBKCFI_43865	AWBRMetXyn19	*Clostridia* bacterium	99.75	2.64e-304	100

Table S4. Some biophysicochemical properties of the characterized xylanases

No	Protein	Instability index	Aliphatic index	Optimum temperature (^oC)	Reference
1	*Streptomyces sp.* S9 xylanase	30.01	73.64	60	86
2	*Bacillus halodurans* S7 xylanase	32.69	78.31	70-75	87
3	*Anoxybacillus sp.* E2 xylanase	38.93	85.27	65	88
4	*Thermomyces lanuginosus*	26.14	62.04	65	89

Table S5. Docking evaluations of the three GH10 xylanases, in reference to XynAS9. The selection process was the lowest binding affinity for each protein. * represents the derivatives of that type of xylooligosaccharides.

PubChem-ID	AWBRMetXyn5 affinity (kcal/mol)	AWBRMetXyn10 affinity (kcal/mol)	AWBRMetXyn19 affinity (kcal/mol)	XynAS9 affinity (kcal/mol)
CID_5107731(X_2)	-6.9	-7	-6.9	-6.6
CID_11305241(X_2^*)	-11.9	-11.9	-11.7	-11.2
CID_131750922(X_2^*)	-8.3	-8.4	-8.3	-8.2
CID_160873(X_2^*)	-7.6	-7.7	-7.7	-7.1
CID_439538(X_2^*)	-8.7	-8.7	-8.4	-7.8
CID_52940183(X_2^*)	-7.7	-7.7	-7.8	-8.1
CID_552964(X_2^*)	-8.6	-8.6	-8.6	-7.8
CID_91746899(X_2^*)	-5.5	-5.5	-5.4	-5.9
CID_10201852(X_3)	-10.5	-10.5	-10.6	-9.2
CID_101857917(X_3^*)	-7.9	-8	-7.6	-7.4
CID_5288379(X_3^*)	-10.6	-10.4	-10.3	-10
CID_5289598(X_3^*)	-10.6	-10.6	-10.4	-9.3
CID_52940105(X_3^*)	-9.1	-9.1	-8.7	-8.7
CID_12444993(X_3^*)	-8.2	-8.2	-7.7	-8.8
CID_91746901(X_3^*)	-6.1	-6	-5.6	-5.8
CID_91873341(X_3^*)	-9.1	-9.4	-9.2	-10.3
**CID_10230811(X_4)**	**-12.4**	**-12.4**	**-12**	**-10.5**
CID_52940205(X_4^*)	-10.6	-10.6	-10.6	-10.4
CID_169447767(X_4^*)	-7.4	-7.1	-6.6	-7.7
CID_91746900(X_4^*)	-7.1	-7	-6	-6.4
CID_5289594(X_4^*)	-12.2	-12.2	-12	-10.7
CID_101656439(X_5)	-11.6	-11.6	-11.2	-10.2
CID_74539951(X_6)	-11.1	-11.1	-10.1	-12.7
CID_600193(X_6^*)	-6.6	-6.7	-6.7	-6.8
CID_91848550(X_7)	-11.5	-11	-10.2	-11.3
CID_600272(X_7^*)	-6.8	-7.1	-6.7	-6.6

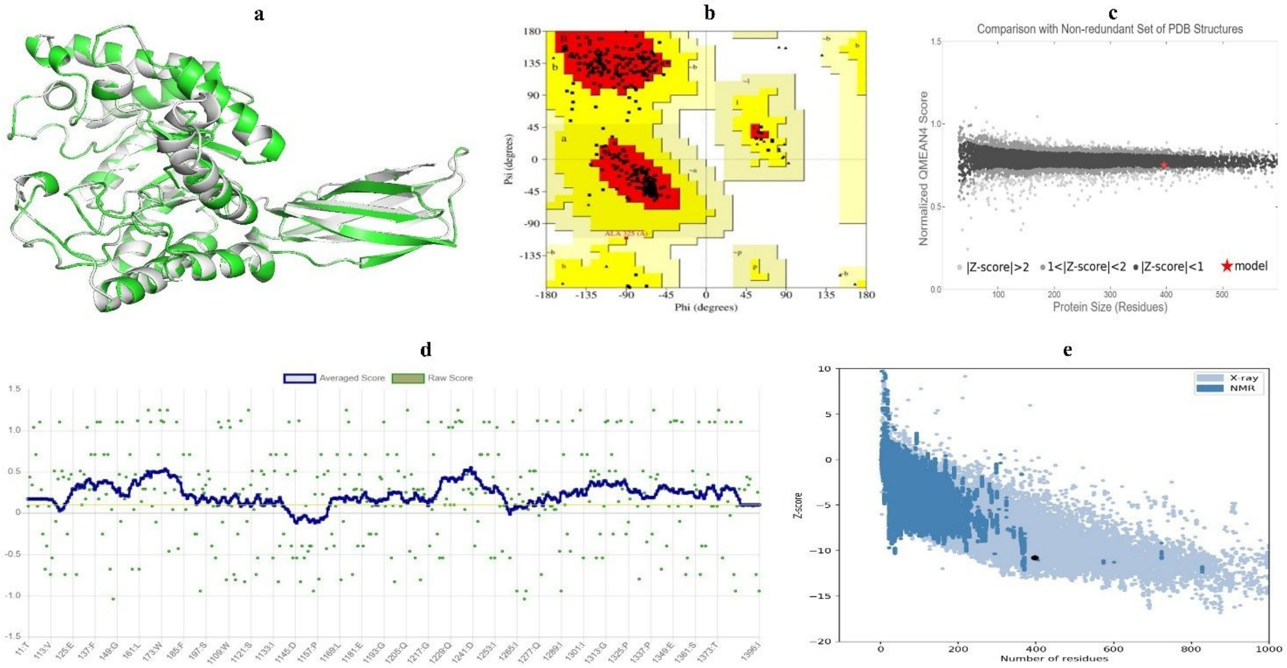

Figure S1. Cross-validation of the predicted structure of AWBRMetXyn5. **a)** the structural alignment of model (green) and template 8B73 (grey). **b)** the ramachandran plot of the model obtained by ProCheck. **c)** the QMEAN graph of the model. **d)** Verify 3D evaluation for compatibility of the model. **e)** Overall quality of the models assessed by ProSA.

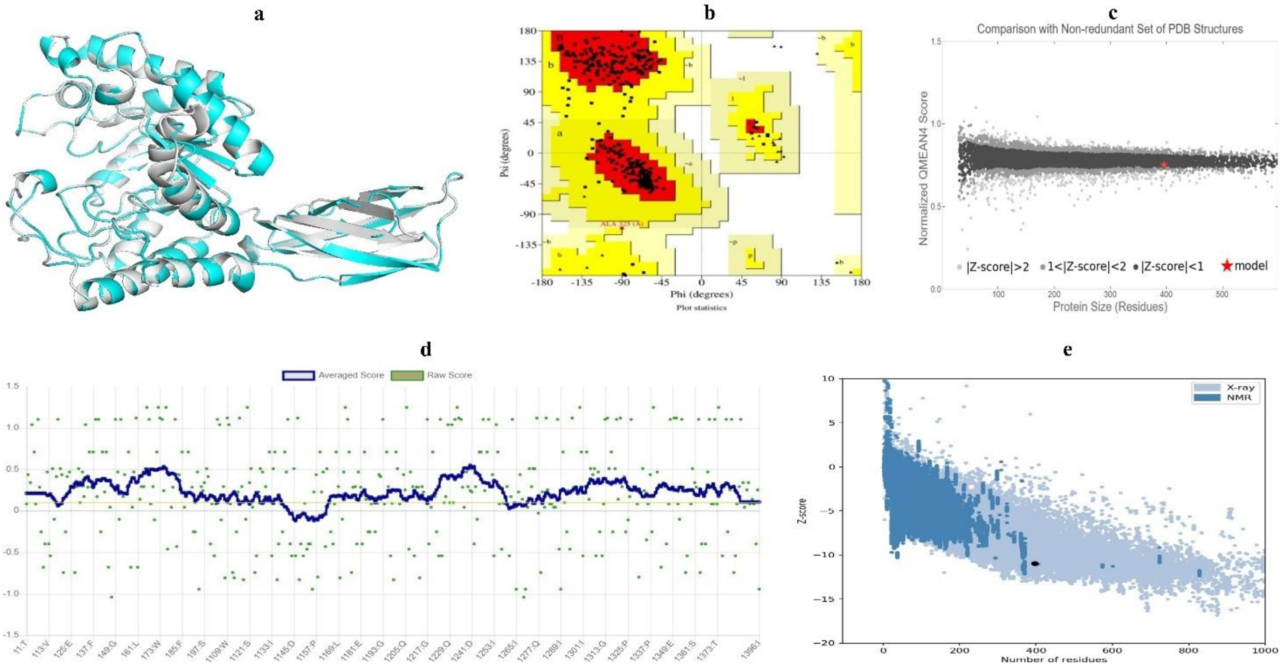

Figure S2. Cross-validation of the predicted structure of AWBRMetXyn10. **a)** the structural alignment of model (cyan) and template 8B73 (grey). **b)** the ramachandran plot of the model obtained by ProCheck. **c)** the QMEAN graph of the model. **d)** Verify 3D evaluation for compatibility of the model. **e)** Overall quality of the models assessed by ProSA.

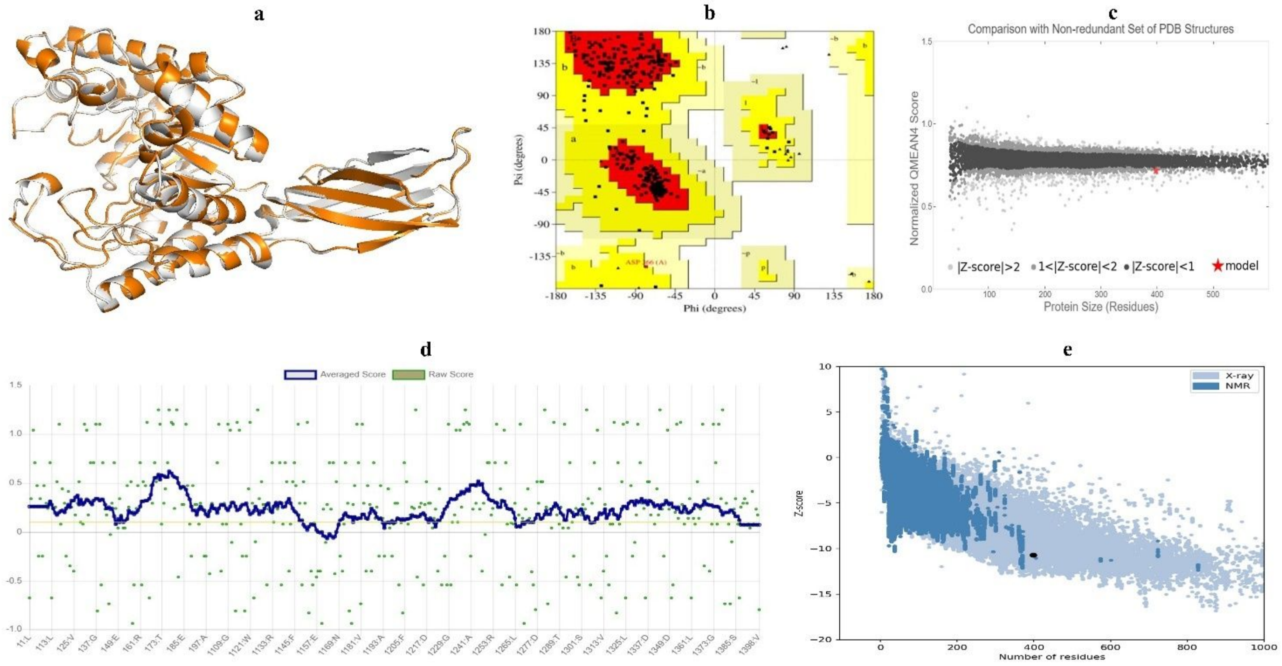

Figure S3. Cross-validation of the predicted structure of AWBRMetXyn19. **a)** the structural alignment of model (orange) and template 8B73 (grey). **b)** the ramachandran plot of the model obtained by ProCheck. **c)** the QMEAN graph of the model. **d)** Verify 3D evaluation for compatibility of the model. **e)** Overall quality of the models assessed by ProSA.
